# Recommendations for empowering early career researchers to improve research culture and practice

**DOI:** 10.1371/journal.pbio.3001680

**Published:** 2022-07-07

**Authors:** Brianne A. Kent, Constance Holman, Emmanuella Amoako, Alberto Antonietti, James M. Azam, Hanne Ballhausen, Yaw Bediako, Anat M. Belasen, Clarissa F. D. Carneiro, Yen-Chung Chen, Ewoud B. Compeer, Chelsea A. C. Connor, Sophia Crüwell, Humberto Debat, Emma Dorris, Hedyeh Ebrahimi, Jeffrey C. Erlich, Florencia Fernández-Chiappe, Felix Fischer, Małgorzata Anna Gazda, Toivo Glatz, Peter Grabitz, Verena Heise, David G. Kent, Hung Lo, Gary McDowell, Devang Mehta, Wolf-Julian Neumann, Kleber Neves, Mark Patterson, Naomi C. Penfold, Sophie K. Piper, Iratxe Puebla, Peter K. Quashie, Carolina Paz Quezada, Julia L. Riley, Jessica L. Rohmann, Shyam Saladi, Benjamin Schwessinger, Bob Siegerink, Paulina Stehlik, Alexandra Tzilivaki, Kate D. L. Umbers, Aalok Varma, Kaivalya Walavalkar, Charlotte M. de Winde, Cecilia Zaza, Tracey L. Weissgerber

**Affiliations:** 1 Department of Psychology, Simon Fraser University, Burnaby, Canada; 2 BIH QUEST Center for Responsible Research, Berlin Institute of Health at Charité–Universitätsmedizin Berlin, Berlin, Germany; 3 Department of Paediatrics and Child Health, Cape Coast Teaching Hospital, Cape Coast, Ghana; 4 Department of Paediatrics and Child Health, School of Medicine, University of Cape Coast, Cape Coast, Ghana; 5 Department of Electronics, Information and Bioengineering, Politecnico di Milano, Milano, Italy; 6 Department of Mathematics, DSI-NRF Center of Excellence in Epidemiological Modelling and Analysis, Stellenbosch University, Stellenbosch, South Africa; 7 Department of Paediatric Endocrinology and Diabetes, Charité—Universitätsmedizin Berlin, Berlin, Germany; 8 West African Centre for Cell Biology of Infectious Pathogens, University of Ghana, Accra, Ghana; 9 Department of Ecology and Evolutionary Biology, Cornell University, Ithaca, New York, United States of America; 10 Society for Conservation Biology, Washington, DC, United States of America; 11 Institute of Medical Biochemistry Leopoldo de Meis, Federal University of Rio de Janeiro, Rio de Janeiro, Brazil; 12 Department of Biology, New York University, New York, New York, United States of America; 13 Kennedy Institute of Rheumatology, University of Oxford, Oxford, United Kingdom; 14 Clemson University, Clemson, South Carolina, United States of America; 15 Center of Agronomic Research, National Institute of Agricultural Technology (IPAVE-CIAP-INTA), Buenos Aires, Argentina; 16 Conway Institute for Biomolecular and Biomedical Research, School of Medicine, University College Dublin, Dublin, Ireland; 17 Non-Communicable Diseases Research Center, Endocrinology and Metabolism Population Sciences Institute, Tehran University of Medical Sciences, Tehran, Iran; 18 New York University Shanghai, Shanghai, China; 19 Sainsbury Wellcome Centre, University College London, London, United Kingdom; 20 Instituto de Investigación en Biomedicina de Buenos Aires, Consejo Nacional de Investigaciones Científicas y Técnicas, Partner Institute of the Max Planck Society, Buenos Aires, Argentina; 21 Department of Psychosomatic Medicine, Center for Internal Medicine and Dermatology, Charité–Universitätsmedizin Berlin, corporate member of Freie Universität Berlin, Humboldt-Universität zu Berlin, and Berlin Institute of Health, Berlin, Germany; 22 Département de Biologie, École Normale Supérieure, CNRS, Institut de Biologie de l’ENS (IBENS), INSERM, Université PSL, Paris, France; 23 Institute of Public Health, Charité—Universitätsmedizin Berlin, Berlin, Germany; 24 Hanse-Wissenschaftskolleg (Institute for Advanced Study), Delmenhorst, Germany; 25 York Biomedical Research Institute, Department of Biology, University of York, York, United Kingdom; 26 Neuroscience Research Center, Charité-Universitätsmedizin Berlin, Germany; 27 Einstein Center for Neurosciences, Berlin, Germany; 28 Lightoller LLC, Chicago Illinois, United States of America; 29 Department of Biological Sciences, University of Alberta, Edmonton, Canada; 30 Movement Disorder and Neuromodulation Unit, Department of Neurology, Charité-Universitätsmedizin Berlin, Berlin, Germany; 31 Retired, Cambridge, United Kingdom; 32 eLife (formerly), Cambridge, United Kingdom; 33 Institute of Biometry and Clinical Epidemiology, Charité—Universitätsmedizin Berlin, Berlin, Germany; 34 Berlin Institute of Health (BIH), Berlin, Germany; 35 ASAPbio, Cambridge, United Kingdom; 36 West African Centre for Cell Biology of Infectious Pathogens, University of Ghana, Accra, Ghana; 37 Francis Crick Institute, London, United Kingdom; 38 Departamento de Química Ambiental, Facultad de Ciencias, Universidad Católica de la Santísima Concepción, Concepción, Chile; 39 Department of Biology, Mount Allison University, New Brunswick, Canada; 40 Center for Stroke Research, Charité—Universitätsmedizin Berlin, Berlin, Germany; 41 California Institute of Technology, Pasedena, California, United States of America; 42 Research School of Biology, The Australian National University, Canberra, Australia; 43 Department of Clinical Epidemiology, Leiden University Medical Center, Leiden, the Netherlands; 44 Directorate of Research Policy, Leiden University Medical Center, Leiden, the Netherlands; 45 Institute for Evidence-Based Healthcare, Bond University, Robina, Australia; 46 Evidence-Based Practice Professorial Unit, Gold Coast Hospital and Health Service, Southport, Australia; 47 NeuroCure Cluster of Excellence Berlin, Germany; 48 School of Science, Western Sydney University, Penrith, Australia; 49 National Centre for Biological Sciences, Tata Institute of Fundamental Research, Bangalore, India; 50 Department of Molecular Cell Biology and Immunology, Amsterdam UMC location VU, Amsterdam, the Netherlands; 51 Centro de Investigaciones en Bionanociencias (CIBION), Consejo Nacional de Investigaciones Científicas y Técnicas (CONICET), Buenos Aires, Argentina

## Abstract

Early career researchers (ECRs) are important stakeholders leading efforts to catalyze systemic change in research culture and practice. Here, we summarize the outputs from a virtual unconventional conference (unconference), which brought together 54 invited experts from 20 countries with extensive experience in ECR initiatives designed to improve the culture and practice of science. Together, we drafted 2 sets of recommendations for (1) ECRs directly involved in initiatives or activities to change research culture and practice; and (2) stakeholders who wish to support ECRs in these efforts. Importantly, these points apply to ECRs working to promote change on a systemic level, not only those improving aspects of their own work. In both sets of recommendations, we underline the importance of incentivizing and providing time and resources for systems-level science improvement activities, including ECRs in organizational decision-making processes, and working to dismantle structural barriers to participation for marginalized groups. We further highlight obstacles that ECRs face when working to promote reform, as well as proposed solutions and examples of current best practices. The abstract and recommendations for stakeholders are available in Dutch, German, Greek (abstract only), Italian, Japanese, Polish, Portuguese, Spanish, and Serbian.

## Abstract 2

Around the world, early-career researchers are working to improve research culture and practice by addressing systemic challenges. This consensus statement provides expert guidance for individuals involved in these projects, and stakeholders who wish to empower the next generation of scientific leadership.

## Introduction

In recent years, the scientific community has been facing a reckoning over the culture and practice of research. What began with concerns about reproducibility [1,2] and waste in biomedical research [3] has expanded to a wide variety of concerns about the academic ecosystem. Critics cite misaligned incentives [4,5], poor working conditions [5,6], and systemic discrimination and bias [7,8] as undermining the discovery and dissemination of new findings. Work to improve research culture and practice is diverse in scope and nature and includes projects focused on themes such as reproducibility, publishing, public involvement, and diversity. This work can take many forms, including initiatives, events, committee activities, and meta-research. Meta-research applies the scientific method to study science itself. This is a powerful approach for identifying problems and developing targeted solutions to improve research conduct [9]. While many stakeholders have highlighted the need for scientific reform, the lack of consensus about what needs to change, how to implement systemic changes, and who should fund and conduct this work creates challenges for those working to improve science.

As the largest and most diverse cohort of scientists [10], ECRs play an important role in science improvement. While definitions differ by country, ECRs include graduate and medical students, young clinical researchers, postdoctoral fellows, and recently appointed independent investigators early in their independent careers. Problems with the scientific system directly affect ECRs, who may have a vested interest in improving the system that they are inheriting. **Box 1** highlights additional reasons why ECR involvement and leadership is important to improving research culture and practice.

**Box 1. Why do we need ECRs to improve science?**

There are many reasons why ECRs are critical players in science improvement.

- **Future leaders:** ECRs are future leaders in the research community. They should be involved in shaping the future of the scientific system.
- **Diverse cohort:** ECRs are a far more diverse cohort than senior scientists [10,16]. Diversity of age, sex, gender identity, sexuality, race, ethnicity, disability, culture, socioeconomic status, language, national origin, and geography bring new perspectives and more creative and generalizable solutions for improving science [17–20]. Diversity decreases with advancing career stage due to prejudicial factors like ableism, classism, racism, and sexism [8].
- **Open to new solutions:** ECRs may be more open to new solutions than senior scientists, who have adopted conventional approaches and succeeded in the existing system [21,22].
- **Optimism:** ECRs may have a yet unchallenged idealism about the scientific enterprise, motivating their reform efforts.
- **“Hands-on” role:** ECRs gain technical skills from collecting and analyzing data with the newest tools that place them at the forefront of advances. ECRs often rely on peer-to-peer networks to learn new skills; therefore, ECRs are critical for implementing best practices in reproducible and open methodology.
- **Time:** While ECR experiences differ, some can invest more time and energy than senior colleagues in science improvement.
- **Majority of the workforce:** ECRs form the majority of the scientific workforce and therefore need to be involved in efforts to implement widespread change, ideally in collaboration with established investigators and other stakeholders.

ECRs have led many successful initiatives to improve research culture and practice. For example, more than 2,000 researchers across 6 continents have received training in reproducible research practices through 25 workshops offered by the ECR-led organization Reproducibility for Everyone [11]. Young Science in Transition is changing incentives by successfully encouraging Dutch universities to adopt PhD evaluation policies that emphasize personal growth and reproducible research practices over bibliometric measures [12]. Black Birders Week, an ECR-initiated social media campaign to raise awareness of Black scientists’ work and the challenges they face, led to the creation of courses and fundraising initiatives for young researchers of color and inspired similar campaigns for other fields [13–15]. **S1 Table** provides additional examples of successful ECR-led initiatives.

This paper summarizes recommendations from an international virtual unconference examining the role of ECRs in catalyzing systemic change in science [8]. Unconferences are participant-driven unconventional conferences designed to maximize informal, stimulating discussions, and networking. The 54 participating experts were invited because of their leading roles in ECR science improvement efforts. Attendees came from 20 countries, were mostly ECRs, and were predominantly working in biomedicine and biology. Details of the unconference format and event were previously reported [8].

Here, we provide 2 sets of recommendations. First, we offer recommendations for early career researchers (ECRs) who are working to improve research culture and practice at a systemic level by launching initiatives, creating peer networks, or advocating for change within organizations. Second, we outline recommendations for stakeholders who wish to support ECRs involved in initiatives to improve research culture and practice. For each of the 6 recommendations, we outline central challenges, suggest potential solutions, and highlight select examples of good practices in the international research community.

**Fig 1:**
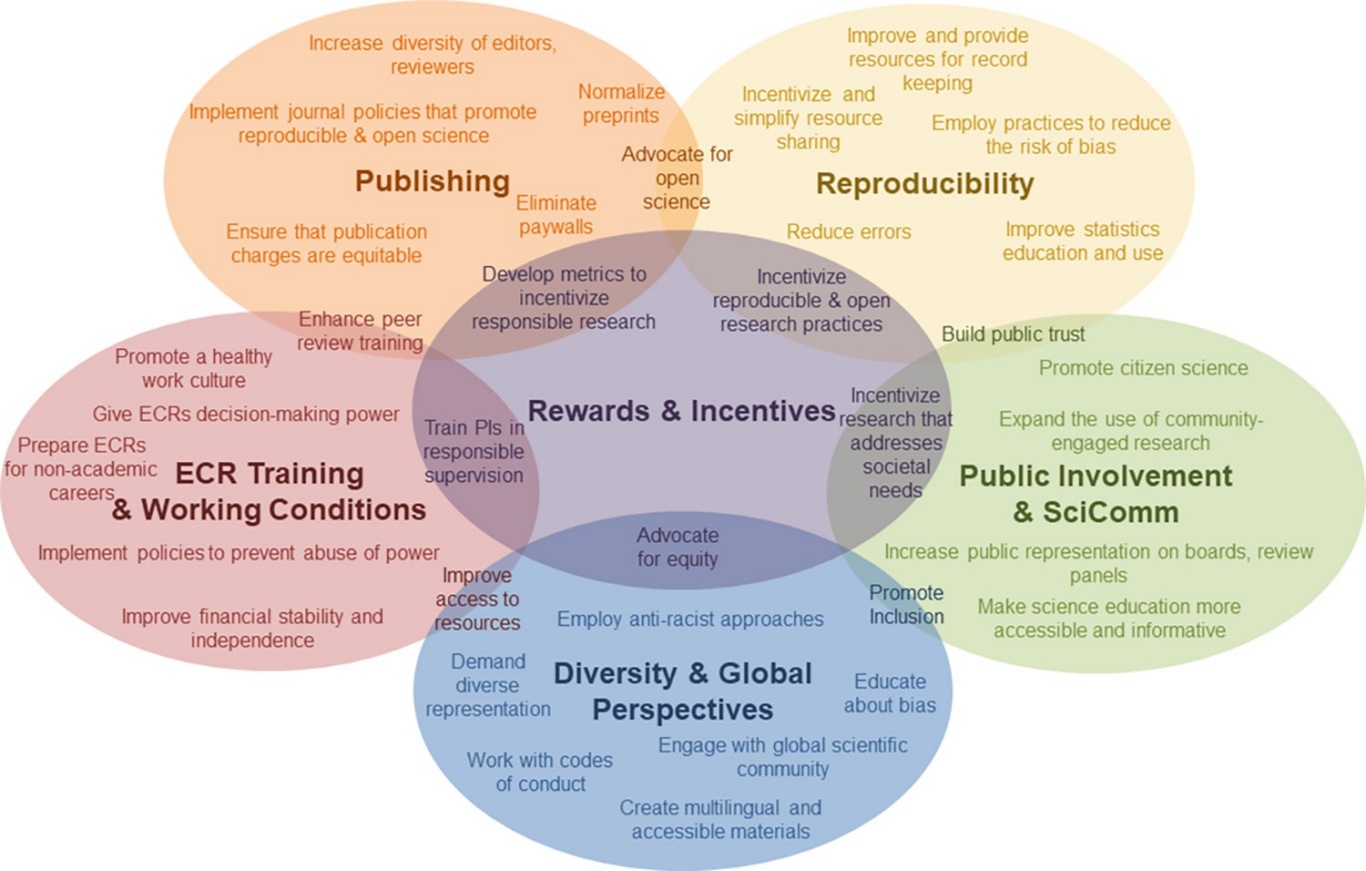
Themes of scientific reform efforts. Common themes of science reform work include publishing, reproducibility, public involvement and science communication, diversity and global perspectives, ECR training and working conditions, and rewards and incentives. This figure provides a general overview of some themes included in each topic and overlapping areas. It is impossible to display all themes and overlapping areas.

Instead of discussing all challenges that ECRs face, or the problems that ECRs encounter when implementing more reproducible practices in their own research, we focus on challenges encountered by the subset of ECRs who are working toward systemic changes. For example, some ECRs are working specifically to promote open science, and the recommendations in this manuscript are designed to help stakeholders support those ECRs working to facilitate change beyond their own research. This might include organizing training to help other scientists implement open science skills, working with publishers to enact policies that encourage authors to use open science practices within their papers, implementing policies that reward open science, changing practices in hiring commissions, faculty evaluations or dissertations, or advising funding agencies on integrating open science practices into funding applications and decisions. While some of these recommendations may also benefit ECRs who are simply implementing new practices in their own research, this is not our focus. **Fig 1** illustrates some of the themes of these initiatives. Unfortunately, we cannot explore all reasons or approaches to improve research culture and practice in this short paper. Instead, we refer readers to excellent reviews cited in the introduction and throughout the recommendations (e.g., [1,3–5,8]).

## Recommendations for ECRs involved in research improvement activities

**Fig 2:**
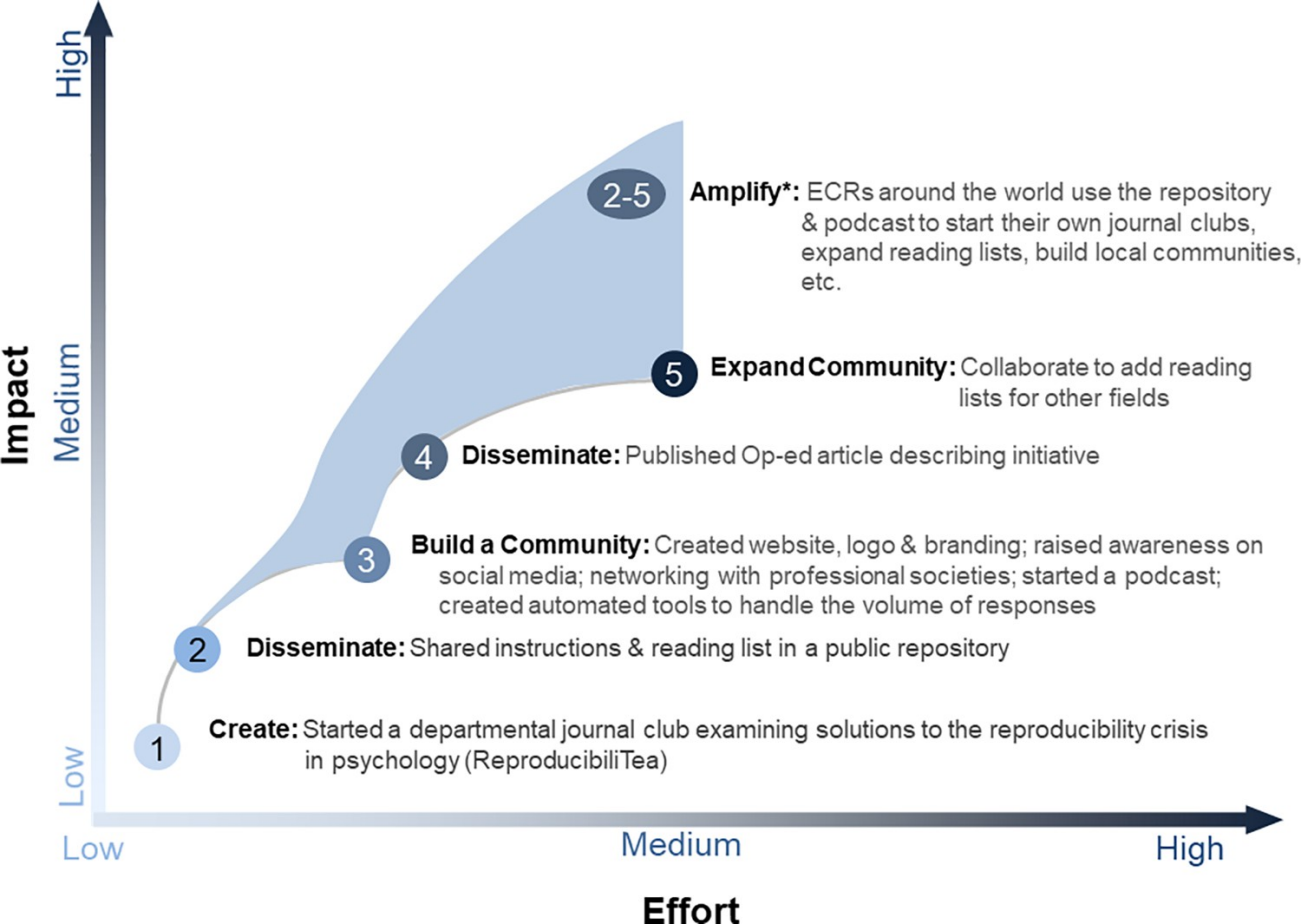
Balancing effort and impact in science reform initiatives. ECRs who are developing an initiative should consider the amount of effort that each stage will require and the potential impact. Actions that allow others to reuse materials to start their own initiatives can amplify impact by expanding the initiative’s reach. This conceptual figure illustrates the relationship between effort and impact for the ReproducibiliTea journal club initiative [23]. The effort and impact values are subjective. Furthermore, it is difficult to determine which specific actions had the greatest impact. Organizers may implement several new approaches simultaneously. Impacts are often delayed, as awareness of initiatives builds over time. Finally, it is important to note that this diagram was based on a successful initiative. Not all initiatives are successful and effort does not always increase impact. Organizers should prioritize actions that they think are most likely to increase impact, then adapt their strategy based on the results. ECR, early career researcher.

This section highlights several recommendations that contribute to the successes of ECR initiatives. **Fig 2** provides a schematic of the stages of project evolution as a function of effort and potential impact. In addition, **S2 Table** provides some ideas for ECRs who are interested in improving research but are unsure where to start. Our online repository (https://osf.io/ad57e) provides additional advice on developing science improvement initiatives, including detailed guidance on topics like understanding organizational structure, developing a communication strategy, and making initiatives sustainable.

### Know what has been done before

ECRs should do their research before starting a new initiative, as they would when planning a new research study. This saves time and builds one’s network by identifying collaborators and allies. Organize structured conversations to identify solutions and attract allies [6]. ECRs may also find materials that they can use or initiatives that they can join or amplify. Understanding why previous efforts did not work is critical—this allows ECRs to anticipate obstacles and develop plans to succeed where others have failed. Developing a new initiative takes time and resource, so look for opportunities to join existing activities that are already having an impact. Joining an existing committee or group may lead to faster results.

### Start with a feasible goal

While ECRs may have an expansive vision and numerous innovative ideas, pursuing many ideas concurrently often leads to frustration, feeling overwhelmed and failure. Starting with stepwise, feasible goals allows the team to refine their approach and materials while building experience and momentum. Once the approach is working, the team can build on their success by expanding to new communities or adding new goals. See **S2 Table** for some ideas on how to get started.

### Collaborate wisely

Initiatives often require skills and expertise in many different areas (e.g., education, policy change, and software development). Look for strong collaborators who have the necessary skills and expertise. The team should also be diverse and representative of the community that the initiative plans to work within. Ensure that team members have time for planned tasks. Add new members as you identify gaps.

### Work toward equity, diversity, and inclusion

Replacing biased, prejudicial, and exclusive systems and behaviors with inclusive alternatives is essential [8]. Initiatives should seek out individuals from minoritized and marginalized groups, remove barriers to participation and ability to thrive, and learn about the impacts of implicit and explicit biases. We encourage all scientists to adopt inclusive and equitable approaches and disseminate these approaches within their initiatives and research environments. Lifelong learning is essential, as equity and inclusion needs and best practices evolve over time. Initiatives should create and disseminate materials in an inclusive and accessible manner. Strategies include using inclusive language, providing closed captioning for recordings, or offering materials in different languages.

### Build a positive and inclusive team dynamic

A code of conduct may help to define expectations for behavior and communication. Ensure that there is a strong moderator to encourage balanced discussion, center, and amplify marginalized voices, and transparently communicate the reasons behind team decisions. Get to know people so that the team dynamic is social and enjoyable. When delegating tasks, agree on roles based on strengths and interests.

### Anticipate concerns or resistance

Learn about the process for making changes at the organization and anticipate objections. Build support by preparing responses to common concerns. Engage with target audiences to understand problems, interests, and solutions. Design achievable and compelling solutions and use feedback to refine these solutions.

### Be persistent

Systemic change takes time. Talk with people at different career stages and within distinct parts of the organization. Do not be discouraged if some of the people approached are not interested. Adapt the team’s strategy to get around roadblocks. There are often many paths to reach a goal; the team only needs to find one path that works.

### Share your work

Identify your target audience before creating materials and develop an effective communication strategy for this audience. You may need a different communication strategy to pitch your initiative to decision makers or potential allies. Choose dissemination platforms that your audience uses (e.g., the Open Science Framework for documents, GitHub for code, and social media for raising awareness). Consider potential barriers to sharing your message, such as language, access to materials, and dissemination outside the team’s networks. Expand your reach by adapting materials for other groups. Collaborate to amplify the efforts of complementary groups with similar goals.

### Plan for sustainability

ECRs are often in transient positions; therefore one must plan for how the initiative will continue after the organizers leave. Systemic change takes time and persistence. The team may need to approach the problem from several angles or collaborate with other groups that approach the problem differently. Sunset the initiative if it is no longer needed.

## Recommendations for stakeholders

**Table 1** summarizes 6 recommendations that stakeholders can implement to support ECR efforts to improve the culture and practice of science. This table and the abstract for the paper are also available in Dutch (S3 Table and S1 Text), German (S4 Table and S2 Text), Greek (abstract only, S3 Text), Italian (S5 Table and S4 Text), Japanese (S6 Table and S5 Text), Polish (S7 Table and S6 Text), Portuguese (S8 Table and S7 Text), Serbian (S9 Table and S8 Text), and Spanish (S10 Table and S9 Text). Each recommendation is accompanied by a series of specific actions. Stakeholders include institutions and departments, funding agencies, scientific societies, journals and publishers, ECR peer communities and allies. Allies, supervisors, and mentors play a special role in empowering ECRs. While these individuals can directly act on recommendations, they can also advocate for ECRs and their science improvement work through other positions that they hold in an organization (e.g., committee work or grant review).

Many, but not all, of these recommendations may require financial investments from stakeholders. Typically, organizations do not provide funding for science improvement activities. Passionate researchers often pursue these activities on a voluntary basis. Funding research improvement activities, however, can pay notable dividends by increasing the quality and transparency of scientific work, and creating a healthier and more productive work culture. A general overview of potential costs may be found in column 3 of **Table 1**, where we have differentiated between actions that likely involve costs ($), actions that may or may not involve costs depending on the setting and implementation ($/−), and actions that typically have low or no costs (−). These ratings are highly subjective, as costs often depend on context and implementation. Adding ECRs to committees, for example, would not require additional funding for volunteer committees that do not require travel for meetings (e.g., institutional or virtual committees). Adding ECRs to committees would require funding if the committees pay members or for national or international committees with in-person meetings. Unfortunately, costs for science improvement activities are often externalized. People donate time because they are passionate about the topic. Once organizations know that people are willing to do the work for free, they see no reason to fund these activities. In our experience, time that passionate ECRs donate when working to improve research culture and practice is a significant externalized cost. Not funding this work undermines and disincentives science improvement efforts.

**Table 1:** Actions that organizations and individuals can take to support ECRs in improving science publishing and research culture.

Recommendation	Supportive actions	Cost	Institutions and departments	Funding agencies	Journals and publishers	Scientific societies	ECR peer communities	Allies, supervisors, and mentors
Provide a path for career progression by rewarding and incentivizing science improvement activities	Create positions for meta-researchers and others working to improve science	**$**	**✔**	**✔**	**✔**	**✔**		**A**
	Reward science improvement activities in hiring and promotion	**-**	**✔**	**✔**	**✔**	**✔**		**A**
	Incorporate science improvement activities into training grant evaluations	**-**	**✔**	**✔**				**A**
	Publish meta-research and science improvement papers (ideally open access)	**$/−**			**✔**			**A**
	Offer awards for science improvement activities	**$/−**	**✔**	**✔**	**✔**	**✔**	**✔**	**A**
Integrate ECRs into decision-making processes	Create advisory groups composed of ECRs and maintain strong dialog with decision-making bodies	**$/−**	**✔**	**✔**	**✔**	**✔**		**A**
	Include ECR representatives on scientific committees; create a welcoming and supportive atmosphere	**$/−**	**✔**	**✔**	**✔**	**✔**		**A**
	Consider combining ECR advisory groups with ECR representatives on committees	**$/−**	**✔**	**✔**	**✔**	**✔**		**A**
Provide ECRs who are skilled in research improvement with resources, funding, and protected time to improve research culture and practice	Create science improvement grants; ensure that ECRs are eligible to apply	**$**	**✔**	**✔**	**✔**	**✔**		**A**
	Create small grants for ECRs who have ideas about improving scientific publishing	**$**		**✔**	**✔**	**✔**		**A**
	Offer logistical or administrative support for ECR initiatives (e.g., a community manager)	**$**	**✔**	**✔**	**✔**	**✔**		**A**
	Publicize programs or outputs valuable to the ECR community	**$/−**	**✔**	**✔**	**✔**	**✔**	**✔**	**✔**
	Offer grants that provide ECRs with protected time for research improvement activities	**$**	**✔**	**✔**		**✔**		**A**
	Encourage ECRs to incorporate science improvement activities into career development plans	**-**	**✔**	**✔**		**✔**		**✔**
Recognize ECRs expertise and amplify their efforts to improve science	Create (online) communities for ECRs working to improve science culture and practices	**$/−**	**✔**	**✔**	**✔**	**✔**	**✔**	**A**
	Train scientists in skills needed to improve science on an individual and systemic level	**$/−**	**✔**	**✔**	**✔**	**✔**	**✔**	**A**
	Provide honest, constructive feedback to help ECRs troubleshoot and refine ideas	**-**	**✔**	**✔**	**✔**	**✔**	**✔**	**✔**
	Use research improvement activities to enhance existing projects	**$/−**	**✔**	**✔**	**✔**	**✔**	**✔**	**✔**
	Work with ECRs to ensure that improvements are sustainable after ECRs move on by integrating changes into standard operating procedures or lab manuals	**-**	**✔**	**✔**	**✔**	**✔**	**✔**	**✔**
	Increase visibility of ECR-led efforts to improve science; give ECRs opportunities to share their research improvement activities with others	**$/−**	**✔**	**✔**	**✔**	**✔**	**✔**	**✔**
Champion efforts to support marginalized ECRs*	Foster a culture of diversity and inclusion	**-**	**✔**	**✔**	**✔**	**✔**	**✔**	**✔**
	Identify and eliminate barriers to full participation	**$/−**	**✔**	**✔**	**✔**	**✔**	**✔**	**✔**
	Enact policies to ensure representation of marginalized groups in leadership positions	**$/−**	**✔**	**✔**	**✔**	**✔**	**✔**	**A**
Support global initiatives to improve research culture and practice	Host virtual or hybrid conferences and networking events, or use formats that enable asynchronous participation (e.g., virtual brainstorming)	**$/−**		**✔**	**✔**	**✔**	**✔**	**A**
	Offer research improvement grants for ECRs in countries or communities with limited research funding	**$**		**✔**		**✔**		**A**
	Scientists from countries where research is comparatively well funded should identify opportunities to amplify the efforts of those with fewer resources	**$/−**	**✔**	**✔**	**✔**	**✔**	**✔**	**✔**
	When adding ECR representatives to committees, include ECRs from countries with limited research funding. Ensure that this diversity is also reflected among non-ECR committee members	**$/−**			**✔**	**✔**	**✔**	**A**

Check marks indicate specific actions that individuals or organizations can take to support and amplify ECR activities to improve science. The letter A denotes actions allies, supervisors, or mentors can advocate for as part of positions that they hold within an organization.

*Individuals and organizations should adopt the 3 recommendations below in all scientific endeavors, including their scientific work and when implementing any actions described in this table. Consult current best practices resources, as diversity, equity, and inclusion practices are context dependent and evolve over time.

ECR, early career researcher.

### Provide a path for career progression by rewarding and incentivizing science improvement activities

Science improvement work is rarely rewarded or incentivized and does not traditionally contribute to career progression. The factors that are rewarded and incentivized determine who secures faculty positions and leadership roles. While universities around the world have departments for fields like biology or epidemiology, only a few centers focus on meta-research or research improvement. Faculty positions in these areas are extremely rare. Meta-research and research improvement activities are not widely valued when hiring faculty, as conventional career advancement criteria prioritize grants and publications in journals with high impact factors. Until meta-research and science improvement activities are rewarded and incentivized, ECRs who excel in these areas will continue to be pushed out of science before securing faculty positions or leadership roles where it is easier to implement systemic changes.

We have suggested 5 specific actions that stakeholders can take to foster a research culture that values science improvement activities alongside more “traditional” outputs (**Table 1**). Some of these specific actions focus on reevaluating existing reward, incentive, and evaluation structures. This includes encouraging individuals or groups that evaluate ECRs, such as hiring and promotion committees and training grants reviewers, to reward systemic efforts to improve science. Institutions and funders will likely need to introduce policy changes that explicitly outline these new criteria and provide training to facilitate implementation. These policy changes should also address equity, diversity, and inclusion (EDI; see also “Champion efforts to support marginalized ECRs” below).

Other specific actions that organizations can take to implement this recommendation involve creating new opportunities to support meta-scientists and others working to improve science, as these activities are essential to the scientific community. For example, creating faculty positions or centers for meta-researchers and other scientists who work on research improvement is essential. Science improvement centers allow researchers to test initiatives at the institution before expanding outward [24]. One such example is Young Science in Transition, an ECR group at the Utrecht University Medical Center, which redesigned PhD evaluation criteria to incorporate personal growth and responsible research practices in addition to publications. These criteria are being adopted by other Dutch graduate schools [12].

*PLOS Biology* has also worked with meta-scientists to launch a meta-research collection [25], which facilitates dissemination by publishing science of science papers in a journal that is often read by traditional scientists. Submitted manuscripts are examined by qualified meta-scientists.

Finally, the Einstein Foundation offers an award for ECRs who are working to improve research [26]. While only one award is given per year, this competition may raise awareness and amplify successful ECR science improvement activities.

### Integrate ECRs into decision-making processes

Organizational hierarchies often exclude ECRs from decision-making roles [27]. Without decision-making power, ECRs struggle to implement change and improve the scientific system. As ECRs make up the majority of the scientific workforce, they should be involved in decision-making at all levels.

Organizations should integrate ECRs at different career stages into their decision-making processes [27] (**Table 1**). Options include creating an early career advisory group, adding ECR representatives to committees or combining both approaches. ECR advisory groups offer a variety of ECR perspectives; however, ECRs are not at the decision-making table. Advisory groups require a consistent, open, and strong dialog with leadership to be effective in setting priorities, refining ideas, and launching initiatives. Alternatively, organizations can also include ECR representatives on committees (e.g., [28]). A disadvantage of this approach is that one ECR voice can easily be suppressed or overlooked. Committees should ensure that the environment is welcoming and inclusive to ECR members. Having at least 2 ECR members per committee offers different perspectives, while providing peer support. Committee work and any costs (e.g., for traveling to meetings) should be distributed and funded equitably to avoid unduly burdening ECRs.

Combining an early career advisory group with ECR committee representatives may be the most effective approach. Advisory group ECRs can serve on committees related to their expertise, while soliciting input from other advisory group members to obtain a broader perspective. Committees selecting ECR members should avoid using career milestones as a proxy for expertise (e.g., applicants must have a PhD). Researchers at the same career stage can have vastly different skills. Organizations should outline necessary skills and ask candidates to explain their experience.

International organizations that add ECRs to committees should include representatives from communities or countries with limited research funding (see also “Champion efforts to support marginalized ECRs” “and “Support global initiatives to improve research culture and practice” below). Diversity at all levels avoids structural inequalities in power structures and creates a more welcoming environment for ECRs from marginalized communities or countries with limited research funding, increasing the likelihood that their perspectives will be considered. ECR committee members should be recruited through internationally advertised open calls, rather than through recommendations from organization members. This may partially level the playing field for those without connections, while extending the organization’s reach.

One example is the eLife Early Career Advisory Group, which includes ECRs from around the world. This group advises eLife on improving scientific publishing and ECR issues and assists with running the eLife Ambassador Program and ECRWednesday webinars. Other organizations have similar advisory roles for ECRs (e.g., Dryad Scientific Advisory Committee, ASAPbio Board of Directors) [29,30].

### Provide ECRs who are skilled in research improvement with resources, funding, and protected time to improve research culture and practice

In our experience, there is a lack of resources and protected time for activities to improve research culture and practice. With notable exceptions (e.g., the Volkswagen Foundation’s Pioneer Projects), most major funders do not offer grants for science improvement work. When federal agencies do offer such grants, the eligibility criteria often exclude postdoctoral fellows and graduate students. ECRs also often lack protected time for research improvement activities, in part because they lack independence and job security. ECRs whose supervisors or mentors do not support their research improvement efforts may confine these activities to evenings and weekends, limiting opportunities to collaborate with stakeholders who are only available during office hours. This problem especially affects those with care responsibilities.

We recommend that stakeholders ensure that opportunities to obtain resources, funding, and protected time for research improvement activities are accessible to ECRs. This includes adapting existing programs to ensure that ECRs can access resources not only as junior partners, but also as leaders. In addition, we recommend that stakeholders create new programs and opportunities to support ECRs in science improvement efforts, such as seed grants for ECR-led initiatives and protected time grants. Finally, stakeholders that train ECRs can encourage ECRs to explore science improvement by incorporating activities to improve research culture and practice into career development plans. By requiring training on topics such as reproducible research practices, organizations support ECRs in developing skills to improve research. Encouraging ECRs to explore the strengths and limitations of existing systems will provide opportunities to develop innovative solutions.

One example of these recommendations in practice is the eLife Innovation Sprint [31], where ECRs and other scientists working on science improvement projects join designers, software developers, and other innovators for a 2-day hackathon to develop new tools to enhance publishing. The host organization benefits from new ideas and opportunities while building relationships with community members. Additionally, the University of Utrecht Open Science Community supports ECRs working to improve science by providing community organizers and faculty ambassadors with protected time to organize open science activities [32].

### Recognize ECRs’ expertise and amplify their efforts to improve science

The perception that ECRs lack the experience and expertise to improve science is sometimes used to justify excluding ECRs from decision-making roles [27]. This inaccurate perception may also cause others to overlook valuable materials and solutions generated by ECRs. While experience and expertise vary among highly diverse ECR cohorts, the many innovative and successful ECR initiatives demonstrate the depth of understanding within the ECR community [33]. Due to their junior status, however, opportunities for ECRs doing this work to gain visibility and implement proposed solutions are limited.

All stakeholders can be an ally by supporting and championing ECR-led efforts to improve research culture and practice. Specific actions for this recommendation focus on fostering an open dialog and collaboration between researchers and stakeholders at all career stages working to improve science. On a “macro” level, organizations can address the perception that ECRs lack the experience and expertise to improve science by publicizing and amplifying successful ECR-led initiatives. This may include organizing symposia or seminar series on ECR initiatives, inviting ECRs to write journal commentaries on their initiatives, or sharing information on initiatives in organizational blogs and newsletters. Organizations can also provide training and networking opportunities, or offer fellowships and hands-on courses in policy change, meta-research [34] or other topics.

Open dialog and collaboration are also critical at a “micro” level within research groups. This allows, for example, integration of science improvement activities into existing research activities, field-testing of solutions on a smaller scale, and implementing procedures to make successful changes sustainable.

For example, the Young Scientists Network of the Academy of Sciences Malaysia design and run responsible conduct of research workshops [35]. Recently, they partnered with the Malaysian Ministry of Higher Education to release an educational module. They are also expanding an instructor training program for the Association of Southeast Asian Nations Young Scientists Network and the regional office of the International Science Council.

### Champion efforts to support marginalized ECRs

Structures, institutions, and a scientific culture that is centered on Europe and North America pose added challenges for ECRs who are members of minoritized and marginalized groups. Examples of the impacts of racism in science include stark disparities in grant funding for Black and Minority Ethnic investigators [7,36]. Persistent sexism contributes to the underrepresentation of people identifying as women in science, technology, engineering, and math (STEM) careers [37,38]. Given the diversity of ECRs, empowering ECRs also means tackling systemic and structural prejudice that make it harder for members of marginalized groups to advocate for themselves and lead reform efforts. The scientific community must recognize that members of these groups face greater obstacles when working to improve science. For example, scientists that hold minoritized and marginalized identities are rarely equitably rewarded for their disproportionate roles in advancing diversity and inclusion [39]. Experiencing prejudicial behavior and structures, without sufficient support, can harm mental health and career progression [40,41]. Eradicating racism and other biases is essential to maintain diversity across all career stages, while ensuring that scientific progress benefits minoritized communities [8].

Organizations, supervisors, mentors, and allies must support efforts to improve EDI in their communities. The interests and memberships of ECR cohorts and marginalized groups frequently intersect; therefore, each action listed in **Table 1** must include measures to empower minoritized and marginalized groups within the ECR community. This involves enacting policies to ensure diverse representation in leadership positions, dismantling structural barriers, and establishing a culture of equity and inclusion to combat and prevent bias and discrimination [42]. Individuals and stakeholders should implement recommendations from resources that outline techniques for creating an inclusive, diverse, and welcoming environment. Resources created by members of marginalized communities can be especially valuable and should be prioritized.

There are many strong examples of projects and initiatives that support ECRs from marginalized communities. The Animal Behaviour Collective reduces socioeconomic barriers to science participation by organizing mentorship opportunities for researchers from traditionally marginalized communities and offering microgrants for animal behavior researchers in financial need [43]. Academics for Black Survival and Wellness is a personal and professional development initiative founded to tackle anti-Black racism in academia and beyond. They provide healing resources for Black folx* and anti-racism training and accountability information for non-Black folx. “Folx” is an altered spelling of “folks” adopted by some groups to include marginalized populations. There are also resources on race and disability [44]. Finally, LGBTQ+ Advocacy in STEM is an online community for LGBTQ+ (lesbian, gay, bisexual, transgender, queer/questioning and others) researchers in STEM professions. The group provides training and resources for LGBTQ+ researchers and allies, with a focus on building safe and inclusive working environments [45].

### Support global initiatives to improve research culture and practice

ECRs working to improve research culture and practice in countries with limited research funding often face added challenges in conducting and publishing their own research, compared to ECRs in countries with higher research funding [46,47]. While economic, geographic, and political differences between countries make it difficult to generalize experiences, the added challenges for ECRs in countries with limited research funding are often amplified when ECRs work to improve science. Conducting research with limited resources (e.g., no laboratory/technical assistants, inflated costs for reagents, and shipping) and infrastructure challenges (e.g., unreliable electricity and/or internet) creates extra work and limits ECRs “free time” for improving research culture and practice.

ECRs working to reform science in countries with limited research funding also experience limited networking opportunities, because of barriers to travel (e.g., lack of travel funding, visa requirements) or poor internet access. In some countries, postdocs and occasionally PIs lack institutional affiliations, which limits local opportunities to initiate systemic change. Language barriers limit access to networking and training opportunities that benefit ECRs working to reforming science. Efforts to improve EDI in academia should increase representation of ECRs from countries with limited research funding, in addition to increasing representation of marginalized groups in countries where research is well funded. However, sometimes these efforts only create the appearance of equity and inclusion (“tokenism”).

Stakeholders can support global initiatives to improve research culture and practice by inviting ECRs from communities or countries with limited research funding to share their activities internationally or collaborating to expand successful initiatives from underrepresented countries to other regions. Virtual or hybrid events bypass the disproportionate obstacles to travel for ECRs in countries with limited research funding [48]. When planning virtual events, facilitating participation from scientists in different time zones is essential. Organizers can create a video archive and plan added networking and moderated discussion times for participants who could not attend the original sessions. One example of a successful initiative that tackles linguistic barriers is PanLingua, a free online tool that uses Google Translate to enable researchers to search for bioRxiv preprints in their own language [49]. A further example that supports community engagement in research is the ECR-led initiative Freshwater Turtiles and Tortises of India on the India Biodiversity Portal. This system aggregates data from citizen scientists while providing open access biodiversity information [50,51].

## Conclusions

ECRs are important stakeholders that are effectively working to catalyze systemic change in research practice and culture. Future efforts should focus on incentivizing and rewarding systemic efforts to improve science culture and practice. This includes providing protected time and faculty positions for individuals working in these areas, amplifying ECR voices and meaningfully incorporating ECRs into decision-making structures. ECRs working on improving science in communities or countries with limited research funding should be supported by organizations with access to greater resources to improve science for all. We hope that the resources shared here will enhance efforts spearheaded by ECRs around the world, while prompting organizations and individuals to take action to support ECRs working to improve science.

## Supporting information

S1 Text Dutch abstract. (DOCX)

S2 Text German abstract. (DOCX)

S3 Text Greek abstract. (DOCX)

S4 Text Italian abstract. (DOCX)

S5 Text Japanese abstract. (DOCX)

S6 Text Polish abstract. (DOCX)

S7 Text Portuguese abstract. (DOCX)

S8 Text Serbian abstract. (DOCX)

S9 Text Spanish abstract. (DOCX)

S1 Table Examples of ECR-led initiatives. ECR, early career researcher; STEM, science, technology, engineering, and medicine.
(DOCX)

S2 Table Ideas for getting started. ECR, early career researcher.
(DOCX)

S3 Table Dutch version of Table 1. ***Acties die organisaties en individuen kunnen nemen om ECRs te ondersteunen bij het verbeteren van wetenschapspublicatie en de wetenschapscultuur*** *Vinkjes geven specifieke acties aan die individuen of organisaties kunnen nemen voor het stimuleren en ondersteunen van ECR-activiteiten om de wetenschap te verbeteren*. De letter A duidt op acties waar collega’s, leidinggevenden of begeleiders voor kunnen pleiten door gebruik te maken van functies die zij binnen een organisatie bekleden. * Individuen en organisaties dienen de drie onderstaande aanbevelingen over te nemen bij alle wetenschappelijke inspanningen, inclusief hun wetenschappelijk werk en bij het uitvoeren van acties die in deze tabel worden beschreven. Raadpleeg de huidige bronnen voor de beste werkwijzen, aangezien praktijken op het gebied van diversiteit, gelijkheid en inclusie contextafhankelijk zijn en over tijd veranderen.
(DOCX)

S4 Table German version of Table 1. **Maßnahmen, die Organisationen und Einzelpersonen ergreifen können, um ECRs bei der Verbesserung von wissenschaftlichen Veröffentlichungen und der Forschungskultur zu unterstützen.** Häkchen kennzeichnen spezifische Maßnahmen, die Einzelpersonen oder Organisationen ergreifen können, um ECR-geleitete-Aktivitäten zur Verbesserung der Wissenschaft zu unterstützen und zu verstärken. Der Buchstabe A kennzeichnet Maßnahmen, für die sich Verbündete, Vorgesetzte oder Mentoren im Rahmen ihrer Positionen innerhalb einer Organisation einsetzen können. * Einzelpersonen und Organisationen sollten die drei folgenden Empfehlungen bei allen wissenschaftlichen Bemühungen einschließlich ihrer wissenschaftlichen Arbeit und bei der Umsetzung der in dieser Tabelle beschriebenen Maßnahmen berücksichtigen. Konsultieren Sie aktuelle Best-Practice-Ressourcen, da Praktiken zur Förderung von Vielfalt, Gleichberechtigung und Integration kontextabhängig sind und sich mit der Zeit weiterentwickeln.
(DOCX)

S5 Table Italian version of Table 1. **Azioni che le organizzazioni e gli individui posso adottare per supportare i ricercatori nelle fasi iniziali della loro carriera nel miglioramento dell’editoria scientifica e della cultura della ricerca.** I simboli di spunta (✔) indicano specifiche azioni che gli individui o le organizzazioni posso adottare per supportare ed amplificare le attività proposte dai giovani ricercatori per migliorare la scienza. La lettera “A” denota le azioni che alleati, supervisori e mentori posso perseguire data la loro posizione all’interno delle organizzazioni di cui fanno parte. *Gli individui e le organizzazioni dovrebbero adottare queste tre raccomandazioni in tutti gli sforzi di ricerca, inclusi il loro lavoro scientifico e quando stiano implementando ognuna delle azioni descritte in questa tabella. Si consultino le risorse riguardo le buone prassi correnti in quanto le pratiche riguardo la diversità, l’equità e l’inclusione dipendono dai contesti ed evolvono nel corso del tempo
(DOCX)

S6 Table Japanese version of Table 1. **科学出版と研究文化の改革に向けてECRを支援するために組織と個人が取るべき行動.**チェックマークは、科学を向上させるためのECR活動を支援・拡大するために、個人または組織が取ることのできる具体的な行動を示す。Aは指導教員、メンターが組織内の役職の一部として提唱できる行動を示す。* 科学的活動を含むすべての活動において、この表に記載された行動を実施する際に、個人および組織は以下の3つの提言を採用すべきである。多様性、公平性、包括性の実践は状況に依存し、時とともに変化するため、現行の最良事例を参照する。
(DOCX)

S7 Table Polish version of Table 1. **Działania, które organizacje i osoby fizyczne mogą podjąć w celu wsparcia ECR w doskonaleniu publikacji naukowych i kultury badawczej.** Zaznaczone pola wskazują konkretne działania, które osoby lub organizacje mogą podjąć w celu wsparcia i wzmocnienia działań ECR w celu poprawy nauki. Litera A oznacza działania, które promotorzy, przełożeni lub mentorzy mogą popierać w ramach stanowisk, które zajmują w organizacji. * Osoby i organizacje powinny przyjąć trzy poniższe zalecenia we wszystkich przedsięwzięciach naukowych, w tym w swojej pracy naukowej i podczas wdrażania wszelkich działań opisanych w tej tabeli. Zapoznaj się z aktualnymi zasobami najlepszych praktyk, ponieważ praktyki w zakresie różnorodności, równości i integracji zależą od kontekstu i ewoluują z czasem.
(DOCX)

S8 Table Portuguese version of Table 1. **Ações que organizações e indivíduos podem adotar para apoiar os ECRs na melhoria da publicação científica e da cultura de investigação.** As marcas de seleção indicam ações específicas que indivíduos ou organizações podem realizar para apoiar e amplificar as atividades de ECRs para melhorar a ciência. A letra A denota ações que aliados, supervisores ou mentores podem defender como parte dos cargos que ocupam dentro de uma organização. * Indivíduos e organizações devem adotar as três recomendações abaixo em todos os empreendimentos científicos, incluindo no seu trabalho científico e ao implementar quaisquer ações descritas nesta tabela. Consulte os recursos atuais de melhores práticas, pois as práticas de diversidade, equidade e inclusão dependem do contexto e evoluem ao longo do tempo.
(DOCX)

S9 Table Serbian version of Table 1. **Aktivnosti koje organizacije i pojedinci mogu da sprovode kako bi podržali mlade istraživače u unapređivanju naučnog izdavaštva i nauke.** Kvačica označava aktivnosti koje pojedinci ili organizacije mogu da sprovedu da bi podržali i uvećali značaj aktivnosti mladih istraživača za unapređivanje nauke. Slovo A označava aktivnosti koje saveznici, supervizori ili mentori mogu da zagovaraju u okviru svojih pozicija unutar organizacija.* Pojedinci i organizacije treba da usvoje tri preporuke u svim svojim naučnim aktivnostima, uključujući naučni rad i kada se primenjuju aktivnosti opisane u ovoj tabeli. Potrebno je konsultovati trenutne resurse o najboljim praksama pošto se prakse diverziteta, jednakosti i inkluzije menjaju tokom vremena i zavise od konteksta.
(DOCX)

S10 Table Spanish version of Table 1. **Acciones que las organizaciones y los individuos pueden tomar para apoyar a los ECR en la mejora de la publicación científica y la cultura de investigación.** Las marcas de verificación indican acciones específicas que los individuos u organizaciones pueden tomar para apoyar y ampliar las actividades de ECR para mejorar la ciencia. La letra A denota acciones por las que los aliados, supervisores o mentores pueden abogar como parte de los puestos que ocupan dentro de una organización.* Las personas y organizaciones deben adoptar las tres recomendaciones en todos los esfuerzos científicos, incluido su trabajo científico y al implementar cualquier acción descrita en esta tabla. Consulte los recursos de mejores prácticas actuales, ya que las prácticas de diversidad, equidad e inclusión dependen del contexto y evolucionan con el tiempo.
(DOCX)

## Abbreviations

ECR: early career researcher
EDI: equity, diversity, and inclusion
LGBTQ+: lesbian, gay, bisexual, transgender, queer/questioning, and others
STEM: science, technology, engineering, and math
