# Supplementary material for: Recommendations for empowering early career researchers to improve research culture and practice
Source: PLoS Biol. 2022 Jul 7;20(7):e3001680. doi: 10.1371/journal.pbio.3001680 (PMC9295962; doi:10.1371/journal.pbio.3001680)
Supplement: S1 Text — (DOCX) [file pbio.3001680.s001.docx]

**Aanbevelingen om Onderzoekers in hun Vroege Loopbaan in staat te stellen Onderzoekscultuur en -praktijk te verbeteren.**

**Abstract:**Onderzoekers die aan het begin van hun loopbaan staan, ook wel early career researchers (ECRs) genoemd, zijn belangrijke aanspoorders van systemische veranderingen in de huidige wetenschapscultuur en -praktijk. In dit artikel vatten we de uitkomsten samen van een virtuele, onconventionele conferentie (*un*conference), waar 54 experts uit 20 landen zijn samen gekomen. Deze experts hebben uitvoerige ervaring met ECR-initiatieven voor het verbeteren van wetenschapscultuur en -praktijk. Samen hebben we twee reeksen van aanbevelingen opgesteld voor (1) ECRs die rechtstreeks betrokken zijn bij initiatieven of activiteiten om de wetenschapscultuur en -praktijk te veranderen, en (2) belanghebbenden die de ECRs zouden willen ondersteunen bij deze inspanningen. Belangrijk is dat deze punten niet alleen van toepassing zijn op ECRs die aspecten van hun eigen werk verbeteren, maar ook op ECRs die werken aan het bevorderen van veranderingen op systeemniveau. In beide reeksen van aanbevelingen leggen we de nadruk op het belang van het stimuleren en beschikbaar stellen van tijd en middelen voor activiteiten die de wetenschap verbetert op systeemniveau, waaronder het betrekken van ECRs in organisatorische besluitvormingsprocessen, en het wegnemen van structurele belemmeringen voor deelname van gemarginaliseerde groepen. Verder benadrukken we obstakels waar ECRs mee worden geconfronteerd bij het bevorderen van hervormingen en presenteren we mogelijke oplossingen en voorbeelden van beste werkwijzen.
