# Supplementary material for: Recommendations for empowering early career researchers to improve research culture and practice
Source: PLoS Biol. 2022 Jul 7;20(7):e3001680. doi: 10.1371/journal.pbio.3001680 (PMC9295962; doi:10.1371/journal.pbio.3001680)
Supplement: S2 Text — (DOCX) [file pbio.3001680.s002.docx]

**Empfehlungen für die Förderung von Nachwuchswissenschaftler:innen zur Verbesserung der Forschungskultur und -praxis**

**Abstract**

Nachwuchswissenschaftler:innen (Early Career Researchers, ECRs) sind wichtige Akteur:innen, die einen systemischen Wandel in der Forschungskultur und -praxis anregen können. Hier fassen wir die Ergebnisse einer virtuellen unkonventionellen Konferenz (*Un*conference) zusammen, an der 54 geladene Expert:innen aus 20 Ländern mit umfassender Erfahrung im Entwickeln von ECR-Initiativen zur Verbesserung der Wissenschaftskultur und -praxis teilgenommen haben. Gemeinsam haben wir zwei Empfehlungspakete für (1) ECRs erarbeitet, welche direkt an Initiativen oder Aktivitäten zur Veränderung der Forschungskultur und -praxis beteiligt sind, und (2) Interessenvertreter:innen, die ECRs bei diesen Bemühungen unterstützen möchten. Wichtig ist, dass diese Punkte für ECRs gelten, die sich für einen Wandel auf systemischer Ebene einsetzen und nicht nur für diejenigen, die Aspekte ihrer eigenen Arbeit verbessern. In beiden Empfehlungspaketen betonen wir, wie wichtig es ist, Anreize zu schaffen sowie Zeit und Ressourcen für Aktivitäten zur Verbesserung der Wissenschaft auf Systemebene bereitzustellen. Zudem sprechen wir uns dafür aus, die ECRs in organisatorische Entscheidungsprozesse einzubeziehen und auf den Abbau struktureller Hindernisse für die Beteiligung marginalisierter Gruppen hinzuarbeiten. Darüber hinaus werden Hindernisse aufgezeigt, mit denen ECRs bei der Förderung von Reformen konfrontiert sind, sowie Lösungsvorschläge und Beispiele für bewährte Praktiken dargestellt.
