# Supplementary material for: Recommendations for empowering early career researchers to improve research culture and practice
Source: PLoS Biol. 2022 Jul 7;20(7):e3001680. doi: 10.1371/journal.pbio.3001680 (PMC9295962; doi:10.1371/journal.pbio.3001680)
Supplement: S3 Text — (DOCX) [file pbio.3001680.s003.docx]

**Recommendations for Empowering Early Career Researchers to Improve Research Culture and Practice**

**Abstract Περίληψη**

Οι νεαροί ερευνητές/τριες (NEς) ενδιαφέρονται ιδιαιτέρως και ηγούνται προσπαθειών με στόχο την καταλυτική και συλλογική αλλαγή της κουλτούρας και των πρακτικών στην έρευνα. Σε αυτό το άρθρο, παρέχουμε την περίληψη, από ένα διαδικτυακό συνέδριο με προσκεκλημένους 54 NEς από 20 χώρες, με μεγάλη εμπειρία και εξειδίκευση σε πρωτοβουλίες με στόχο τη βελτίωση της κουλτούρας και των πρακτικών στην επιστημονική κοινότητα. Μαζί, συγγράψαμε δυο προτάσεις για (1) ΝΕς που ενδιαφέρονται να συνεισφέρουν ενεργά σε πρωτοβουλίες ή δραστηριότητες με στόχο την αλλαγή της ερευνητικής πρακτικής και κουλτούρας και (2) για ενδιαφερόμενους που θα ήθελαν να υποστηρίξουν στο έργο και τις προσπάθειες των ΝΕς. Οι προτάσεις αυτές αφορούν ΝΕς που προωθούν στην αλλαγή σε συλλογικό και όχι μόνο ατομικό επίπεδο. Και στις δύο προτάσεις μας, υπογραμμίζουμε την βαρύνουσα σημασία που έχουν τα κίνητρα, ο χρόνος και η παροχή πόρων για τις συλλογικές δραστηριότητες με στόχο την βελτίωση της επιστήμης. Τέτοιες δραστηριότητες συμπεριλαμβάνουν τους ΝΕς σε διαδικασίες λήψης αποφάσεων και προάγουν την άρση των φραγμών που κάποιες ομάδες ΝΕς αντιμετωπίζουν. Επιπλέον, παραθέτουμε τα γενικότερα εμπόδια που οι ΝΕς αντιμετωπίζουν και προτείνουμε παραδείγματα και λύσεις.
