# Supplementary material for: Recommendations for empowering early career researchers to improve research culture and practice
Source: PLoS Biol. 2022 Jul 7;20(7):e3001680. doi: 10.1371/journal.pbio.3001680 (PMC9295962; doi:10.1371/journal.pbio.3001680)
Supplement: S4 Text — (DOCX) [file pbio.3001680.s004.docx]

**Raccomandazioni per consentire ai ricercatori nelle fasi iniziali della loro carriera di migliorare la cultura e le pratiche nella ricerca**

**Sommario**

I ricercatori nelle fasi iniziali della loro carriera (qui chiamati “giovani ricercatori”) sono gli attori principali per guidare il cambiamento sistemico nella cultura e nelle pratiche della ricerca. Qui, riassumiamo i risultati ottenuti durante una conferenza virtuale non convenzionale, che ha riunito 54 esperti invitati provenienti da 20 paesi con una vasta esperienza in iniziative focalizzate sui giovani ricercatori e progettate per migliorare la cultura e le pratiche della scienza. Insieme agli esperti abbiamo redatto due serie di raccomandazioni rivolte:

1. ai giovani ricercatori direttamente coinvolti in iniziative o attività volte a promuovere un cambiamento nella cultura e nelle pratiche della ricerca;
2. alle parti interessate che desiderano supportare i ricercatori in questi sforzi.

È importante sottolineare che queste raccomandazioni si applicano sia ai ricercatori che si vogliono impegnare per un cambiamento a livello sistemico sia a quelli che intendono migliorare gli aspetti relativi al proprio lavoro.

In entrambe le serie di raccomandazioni, sottolineiamo l'importanza di incentivare le attività di miglioramento della scienza a livello di sistema assegnandovi tempo e risorse, includere i giovani ricercatori nei processi decisionali organizzativi, e lavorare per smantellare le barriere strutturali alla partecipazione per i gruppi tradizionalmente più emarginati. Evidenziamo inoltre gli ostacoli che i giovani ricercatori devono affrontare quando lavorano per promuovere questa riforma, nonché alcune soluzioni proposte e degli esempi di buone prassi attualmente impiegate.
