# Supplementary material for: Recommendations for empowering early career researchers to improve research culture and practice
Source: PLoS Biol. 2022 Jul 7;20(7):e3001680. doi: 10.1371/journal.pbio.3001680 (PMC9295962; doi:10.1371/journal.pbio.3001680)
Supplement: S5 Text — (DOCX) [file pbio.3001680.s005.docx]

**研究の文化と実践をより良くするための、早期キャリア研究者強化に関する提言**

要旨

早期キャリア研究者 (Early career researchers, ECRs) は研究の文化と実践のシステムの改革を促す重要な関係者である。私たちは研究の文化と実践を向上させることを目的としたECRの取り組みに豊富な経験を持つ、20カ国54名の招待された専門家が集まった、ユニークな仮想型会議（*un*conference）の成果を要約してご紹介する。私たちは (1) 研究の文化と実践を改革するための取り組みや活動に直接携わるECRと、(2) こうした取り組みにおいてECRを支援したいと考える関係者のために、2つの提言を作成した。重要なことは、これらの提言は、自身の仕事に関する側面を改革するECRだけでなく、システムレベルでの改革を促進するECRにも適用されることである。2つの提言において、私たちは組織の意思決定プロセスにECRを参加させ、社会から疎外されたグループの参加を阻む構造的障害を取り除くことを含むシステムレベルでの改革に対する時間と資源を提供し動機を与える重要さを強調する。さらに、ECRが改革を推進する際に直面する障害並びに提案された解決策、現在の最善の実践例について紹介する。
