# Supplementary material for: Recommendations for empowering early career researchers to improve research culture and practice
Source: PLoS Biol. 2022 Jul 7;20(7):e3001680. doi: 10.1371/journal.pbio.3001680 (PMC9295962; doi:10.1371/journal.pbio.3001680)
Supplement: S6 Text — (DOCX) [file pbio.3001680.s006.docx]

**Zalecenia dotyczące wzmocnienia pozycji początkujących naukowców w celu poprawy kultury i praktyki badawczej**

**Streszczenie**

Naukowcy na wczesnym etapie kariery (Early Career Researchers (ECRs)) są ważnymi osobami prowadzącymi wysiłki na rzecz wprowadzania systemowych zmian w kulturze i praktyce badawczej.

W naszej pracy podsumowujemy wyniki wirtualnej, niekonwencjonalnej konferencji (“unconference”), która zgromadziła 54 zaproszonych ekspertów z 20 krajów. Eksperci Ci posiadają bogate doświadczenie w inicjatywach początkujących naukowców mających na celu poprawę kultury i praktyk naukowych. Wspólnie opracowaliśmy dwa zestawy zaleceń dotyczących (1) młodych naukowców, bezpośrednio zaangażowanych w inicjatywy lub działania mające na celu zmianę kultury i praktyk badawczych oraz (2) zainteresowanych stron, które chcą wspierać początkujących naukowców w tych wysiłkach. Co ważne, punkty te odnoszą się do młodych naukowców działających na rzecz promowania zmian na poziomie systemowym, a nie tylko tych, które poprawiają aspekty ich własnej pracy. W obu zestawach zaleceń podkreślamy znaczenie zachęcania i zapewniania czasu i zasobów na działania na rzecz poprawy nauki na poziomie systemowym, w tym udziału początkujących naukowców w  procesach decyzyjnych, oraz pracy nad zlikwidowaniem strukturalnych barier uczestnictwa dla grup marginalizowanych. Ponadto wyjaśniamy przeszkody, jakie napotykają początkujący naukowcy podczas pracy nad promowaniem reform, a także proponujemy rozwiązania i przykłady istniejących już najlepszych praktyk.
