# Supplementary material for: Recommendations for empowering early career researchers to improve research culture and practice
Source: PLoS Biol. 2022 Jul 7;20(7):e3001680. doi: 10.1371/journal.pbio.3001680 (PMC9295962; doi:10.1371/journal.pbio.3001680)
Supplement: S7 Text — (DOCX) [file pbio.3001680.s007.docx]

**Recomendações para o Empoderamento de Investigadores em Início de Carreira para Melhorar a Cultura e a Prática Científica**

**Abstrato**

Investigadores em início de carreira (ECRs) são intervenientes importantes que lideram esforços para acelerar mudanças sistémicas na cultura e prática da investigação científica. Aqui, resumimos os resultados de uma conferência virtual não convencional (*un*conference), que reuniu 54 especialistas de 20 países com ampla experiência em iniciativas de ECR destinadas a melhorar a cultura e a prática científica. Juntos, elaborámos dois conjuntos de recomendações para (1) ECRs diretamente envolvidos em iniciativas ou atividades para mudar a cultura e a prática de investigação e (2) intervenientes que desejam apoiar os ECRs nesses esforços. É importante salientar que estes pontos se aplicam aos ECRs que trabalham para promover mudanças a um nível sistémico, e não para melhorar apenas aspetos do seu próprio trabalho. Em ambos os conjuntos de recomendações, destacamos a importância do incentivo e disponibilidade de tempo e recursos para atividades de melhoria científica a nível dos sistemas, incluindo ECRs em processos de tomada de decisão organizacional, e trabalhar para destruir barreiras estruturais à participação de grupos marginalizados. Destacamos ainda os obstáculos que os ECRs enfrentam ao trabalhar para promover a reforma, bem como soluções propostas e exemplos das melhores práticas atuais.
