# Supplementary material for: Recommendations for empowering early career researchers to improve research culture and practice
Source: PLoS Biol. 2022 Jul 7;20(7):e3001680. doi: 10.1371/journal.pbio.3001680 (PMC9295962; doi:10.1371/journal.pbio.3001680)
Supplement: S8 Text — (DOCX) [file pbio.3001680.s008.docx]

**Preporuke za osnaživanje mladih istraživača radi unapređenja istraživačke kulture i prakse**

**Apstrakt**

Mladi istraživači su bitni interesna strana čiji su napori suštinski za pokretanje sistemskih promena u istraživačkoj kulturu i praksi. U ovom radu je dat sažetak rezultata virtualne nekonvencionalne konferencije (ne-konferencije) koja je okupila 54 pozvanih eksperata iz 20 zemalja sa opsežnim iskustvom u incijativama mladih istraživača osmišljenim za unapređivanje naučne kulture i prakse. Zajedno s njima smo sačinili dva skupa preporuka za (1) mlade istraživače koji su neposredno uključeni u inicijative i aktivnosti za promenu istraživačke kulture i prakse, kao i za (2) interesne strane koje žele da podrže mlade istraživače u njihovim nastojanjima.

Bitno je naglasiti da su ove stavke primenjive na mlade istraživače koji rade na promociji sistemskih promena, a ne samo na unapređivanju onih aspekata koji se tiču njihovog sopstvenog rada. U oba skupa preporuka, potcrtali smo u kojoj meri je bitno davanje podsticaja, obezbeđivanje vremena i resursa za aktivnosti za sistemsko unapređivanje nauke, uključivanje mladih istraživača u procese organizacinog donošenja odluka i rad na uklanjanju strukturnih prepreka koje stoje na putu učešća marginalnih grupa. U nastavku su naglašene prepreke sa kojima se mladi istraživači susreću u svom radu na promociji reforme, kao i preporučena rešenja i primeri trenutnih najboljih praksi.
