# Supplementary material for: Recommendations for empowering early career researchers to improve research culture and practice
Source: PLoS Biol. 2022 Jul 7;20(7):e3001680. doi: 10.1371/journal.pbio.3001680 (PMC9295962; doi:10.1371/journal.pbio.3001680)
Supplement: S9 Text — (DOCX) [file pbio.3001680.s009.docx]

**Recomendaciones para Empoderar a los Investigadores de Carrera Temprana orientada a Mejorar la Cultura y la Práctica en la Investigación**

**Resumen**

Los investigadores de carrera temprana (ECR, por sus siglas en inglés) son partes interesadas importantes que lideran los esfuerzos para catalizar el cambio sistémico en la cultura y la práctica en la investigación. Aquí, resumimos los resultados de una conferencia virtual no convencional (*no-*conferencia), que reunió a 54 expertos invitados de 20 países con amplia experiencia en iniciativas ECR diseñadas para mejorar la cultura y la práctica de la ciencia. Juntos, redactamos dos conjuntos de recomendaciones para (1) los ECR directamente involucradas en iniciativas o actividades para cambiar la cultura y la práctica en la investigación, y (2) las partes interesadas que deseen apoyar a los ECR en estos esfuerzos. Es importante destacar que estos puntos se aplican a los ECR que trabajan para promover el cambio a nivel sistémico, no solo a aquellos que mejoran aspectos de su propio trabajo. En ambos conjuntos de recomendaciones, subrayamos la importancia de incentivar y proporcionar tiempo y recursos para las actividades de mejora de la ciencia a nivel de sistemas, incluyendo a los ECR en los procesos de toma de decisiones organizacionales, y trabajando para desmantelar las barreras estructurales a la participación de los grupos marginados. Destacamos además los obstáculos que enfrentan los ECR cuando trabajan para promover la reforma, así como soluciones propuestas y ejemplos de las mejores prácticas actuales.
