# Supplementary material for: Recommendations for empowering early career researchers to improve research culture and practice
Source: PLoS Biol. 2022 Jul 7;20(7):e3001680. doi: 10.1371/journal.pbio.3001680 (PMC9295962; doi:10.1371/journal.pbio.3001680)
Supplement: S1 Table — ECR, early career researcher; STEM, science, technology, engineering, and medicine. (DOCX) [file pbio.3001680.s010.docx]

| **Type of Initiative** | **Example** | **Description** |
| --- | --- | --- |
| ECR advocacy | [Future of Research](https://www.futureofresearch.org/) | ECR advocacy organization; provides evidence-based resources to improve the scientific research enterprise |
|  | [COI-Network (Germany)](http://www.interessenkonflikte.com/) | Medical student advocacy group; works with institutional leadership to ask instructors to include conflicts of interest (COI) in curricula and disclose COI while teaching |
| Training | [Peerspectives](https://iph.charite.de/en/academic_programs/phd_in_health_data_sciences/peerspectives/) | Peer-review training program for PhD students and recently graduated postdocs |
| Online platforms, resources and blogs | [ecrLife](https://ecrlife.org/) | Blog for ECRs to share stories, ideas and experiences about their science journey |
|  | [ecrCentral](https://ecrcentral.org/) | Community-driven platform for sharing and finding funding opportunities for ECRs |
|  | [The Black Hole](https://www.universityaffairs.ca/opinion/the-black-hole/) | *University Affairs* magazine column on issues affecting ECRs |
| Journal clubs | [ReproducibiliTea](https://reproducibilitea.org/) | ECR-driven initiative; helps researchers create local journal clubs to discuss ways to improve science, reproducibility, and Open Science |
|  | [PREreview](https://www.prereview.org/) | ECR-driven platform for crowdsourcing preprint reviews; |
| Community building to amplify impact | [eLife Ambassadors](https://elifesciences.org/inside-elife/263fa4d1/elife-ambassadors-a-celebration-of-the-second-cohort) | ECR program to create an advocacy network & promote responsible behaviours in science [1]; |
|  | [#BlackinSTEM social media campaigns](https://www.usatoday.com/story/life/2020/08/04/blackinneuro-blackinchem-can-hashtags-help-black-scientists-build-community-spotlight-excellence/5541431002/) | ECR-driven hashtag campaigns to showcase minoritized and marginalized groups in science and draw attention to systemic racism [2]; |

**S1 Table:** Examples of ECR-led initiatives

Abbreviations: ECR, early career researcher; STEM, science, technology, engineering and medicine

**References:**

1. eLife, *eLife Ambassadors: A celebration of the second cohort,* eLife, 2020.

2. Mallenbaum, C*., #BlackBirdersWeek, #BlackInNeuro: Black scientists, physicians are using hashtags to uplift*, in *USA Today*. 2020.
