# Supplementary material for: Recommendations for empowering early career researchers to improve research culture and practice
Source: PLoS Biol. 2022 Jul 7;20(7):e3001680. doi: 10.1371/journal.pbio.3001680 (PMC9295962; doi:10.1371/journal.pbio.3001680)
Supplement: S2 Table — ECR, early career researcher. (DOCX) [file pbio.3001680.s011.docx]

| **Activity** | **Impact** | **Resources & Examples** |
| --- | --- | --- |
| Join a community for training and opportunities | Learn skills and meet like-minded individuals | [**ASAPbio Community**](https://asapbio.org/asapbio-community) |
| Join a committee or advocate for ECR representation on a committee | Increase the number of ECRs in decision-making roles | **Research Culture: Why scientific societies should involve more early-career researchers** [1] |
| Participate in a public outreach event | Raise awareness of science and why it is important | [**Skype a Scientist**](https://www.skypeascientist.com/) |
| Write a blog or opinion piece for an existing platform | Highlight important issues and opinions | [**The OpED Project**](https://www.theopedproject.org/) |
| Organize Reproducibility for Everyone workshop for your lab, department or institution | Train others on best practices for reproducible science | [**Reproducibility for Everyone**](https://repro4everyone.org/pages/host/) |
| Start or join a social media hashtag campaign | Activate a network of like-minded researchers interested in a specific topic | [**#LabWasteDay**](https://ecrsustainable.wixsite.com/sustainablescience/labwasteday) |
| Organize a preprint journal club | Discuss new findings and draw attention to preprint manuscripts; post your review in the preprint’s comment section or to a public review platform | **Point of View: Journal clubs in the time of preprints** [2] |
| Create a committee for ECRs at your institution | Generate a political body to advocate for ECR interests | [**UBC Postdoctoral Association**](https://blogs.ubc.ca/ubcpda/) |
| Organize structured conversations to identify solutions to problems | Identify like-minded individuals who share your priorities for change | **Shaping the Future of Research: a perspective from junior scientists [3**] |
| Abbreviations: ECR, early career researcher | | |

**Supplementary Table S2:** Ideas for getting started

**References:**

1. Bankston, A., et al., Research Culture: *Why scientific societies should involve more early-career researchers*. Elife, 2020. 9: p. e60829.

2. Avasthi, P., A. Soragni, and J.N. Bembenek, *Point of View: Journal clubs in the time of preprints*. Elife, 2018. 7: p. e38532.

3. McDowell, G.S., et al., *Shaping the future of research: a perspective from junior scientists*. F1000Research, 2014. 3.
