# Supplementary material for: Recommendations for empowering early career researchers to improve research culture and practice
Source: PLoS Biol. 2022 Jul 7;20(7):e3001680. doi: 10.1371/journal.pbio.3001680 (PMC9295962; doi:10.1371/journal.pbio.3001680)
Supplement: S3 Table — Acties die organisaties en individuen kunnen nemen om ECRs te ondersteunen bij het verbeteren van wetenschapspublicatie en de wetenschapscultuur Vinkjes geven specifieke acties aan die individuen of organisaties kunnen nemen voor het stimuleren en ondersteunen van ECR-activiteiten om de wetenschap te verbeteren. De letter A duidt op acties waar collega’s, leidinggevenden of begeleiders voor kunnen pleiten door gebruik te maken van functies die zij binnen een organisatie bekleden. * Individuen en organisaties dienen de drie onderstaande aanbevelingen over te nemen bij alle wetenschappelijke inspanningen, inclusief hun wetenschappelijk werk en bij het uitvoeren van acties die in deze tabel worden beschreven. Raadpleeg de huidige bronnen voor de beste werkwijzen, aangezien praktijken op het gebied van diversiteit, gelijkheid en inclusie contextafhankelijk zijn en over tijd veranderen. (DOCX) [file pbio.3001680.s012.docx]

**Aanbevelingen om Onderzoekers in hun Vroege Loopbaan in staat te stellen Onderzoekscultuur en -praktijk te verbeteren.**

| **Aanbeveling**​ | **Ondersteunende Acties**​ | **Kosten** | **Instituten & Afdelingen** | **Financierings-instanties** | **Tijdschriften &**​ **Uitgevers**​ | **Wetenschappelijke Verenigingen**​ | **ECR Gemeenschappen**​ | **Collega’s, Leidinggevenden & Begeleiders**​ |
| --- | --- | --- | --- | --- | --- | --- | --- | --- |
| Bied een pad voor loopbaanontwikkeling door activiteiten die de wetenschap verbeteren te belonen en te stimuleren | Creëer banen voor meta-onderzoekers en anderen die werken aan het verbeteren van de wetenschap | **$** | **✔**​ | **✔**​ | **✔**​ | **✔**​ | ​ | **​A** |
|  | Beloon verleden activiteiten die de wetenschap verbeteren bij het aannemen van nieuwe medewerkers of bij promoties | **-** | **✔**​ | **✔**​ | **✔**​ | **✔**​ | ​ | **​A** |
|  | Neem activiteiten die de wetenschap verbeteren op in de evaluaties van opleidingsbeurzen | **-** | **✔**​ | **✔**​ | ​ | ​ | ​ | **​A** |
|  | Publiceer meta-onderzoek en artikelen die beschrijven hoe de wetenschap kan worden verbeterd (bij voorkeur open access) | **$/-** | ​ | ​ | **✔**​ | ​ | ​ | **​A** |
|  | Bied prijzen aan voor activiteiten die de wetenschap verbeteren | **$/-** | **✔**​ | **✔**​ | **✔**​ | **✔**​ | **✔** | **​A** |
| Includeer ECRs bij besluitvormingsprocessen | Creëer adviesgroepen van ECRs en onderhoud een gesprek tussen hen en besluitvormende organen | **$/-** | **✔**​ | **✔**​ | **✔**​ | **✔**​ | ​ | **​A** |
|  | Neem ECR-vertegenwoordigers op in wetenschappelijke comités; cre​ëer een gastvrije en ondersteunende sfeer | **$/-** | **✔**​ | **✔**​ | **✔**​ | **✔**​ | ​ | **​A** |
|  | Overweeg om ECR-adviesgroepen te combineren met ECR-vertegenwoordigers in commissies | **$/-** | **✔**​ | **✔**​ | **✔**​ | **✔**​ | ​ | **​A** |
| Voorzie ECRs die bekwaam zijn in het verbeteren van de wetenschap van middelen, financiering en tijd om de wetenschapscultuur en -praktijk te verbeteren | Creëer subsidies voor wetenschappelijke verbetering; zorg ervoor dat ECRs in aanmerking komen om zich aan te melden | **$** | **✔**​ | **✔**​ | **✔**​ | **✔**​ | ​ | **​A** |
|  | Creëer kleine subsidies voor ECRs die ideeën hebben om het huidige proces van wetenschapspublicaties te verbeteren | **$** | ​ | **✔**​ | **✔**​ | **✔**​ | ​ | **​A** |
|  | Bied logistieke of administratieve steun aan ECR-initiatieven (bijv. een community manager) | **$** | **✔**​ | **✔**​ | **✔**​ | **✔**​ | ​ | **​A** |
|  | Publiceer programma’s of uitkomsten die waardevol zijn voor de ECR-gemeenschap | **$/-** | **✔**​ | **✔**​ | **✔**​ | **✔**​ | **✔** | **✔**​ |
|  | Bied subsidies aan die ECRs tijd biedt voor activiteiten om wetenschap te verbeteren | **$** | **✔**​ | **✔**​ | ​ | **✔**​ | ​ | **​A** |
|  | Stimuleer ECRs om activiteiten voor verbetering van de wetenschap op te nemen in hun loopbaanontwikkelingsplan | **-** | **✔**​ | **✔**​ | ​ | **✔**​ | ​ | **✔**​ |
| Erken de expertise van ECRs en versterk hun inspanningen om wetenschap te verbeteren  ​  ​ | Creëer (online) gemeenschappen voor ECRs die werken aan het verbeteren van de wetenschapscultuur en -praktijken | **$/-** | **✔**​ | **✔**​ | **✔**​ | **✔**​ | **✔**​ | **​A** |
|  | Leid wetenschappers op in vaardigheden die nodig zijn om de wetenschap op individueel en systemisch niveau te verbeteren | **$/-** | **✔**​ | **✔**​ | **✔**​ | **✔**​ | **✔**​ | **​A** |
|  | Geef eerlijke, opbouwende feedback om ECRs te helpen bij het oplossen en verfijnen van ideeën | **-** | **✔**​ | **✔**​ | **✔**​ | **✔**​ | **✔**​ | **✔**​ |
|  | Gebruik activiteiten om de wetenschap te verbeteren om bestaande projecteren te verbeteren | **$/-** | **✔**​ | **✔**​ | **✔**​ | **✔**​ | **✔**​ | **✔**​ |
|  | Werk samen met ECRs om ervoor te zorgen dat verbeteringen duurzaam zijn nadat ECRs verder zijn in hun carrière door veranderingen op te nemen in laboratoriumhandleidingen of aan te nemen als standaardprocedures. | **-** | **✔**​ | **✔**​ | **✔**​ | **✔**​ | **✔**​ | **✔**​ |
|  | Vergroot de zichtbaarheid van de door ECRs geleide inspanningen om de wetenschap te verbeteren; geef ECRs kansen om hun activiteiten met anderen te delen ​ | **$/-** | **✔**​ | **✔**​ | **✔**​ | **✔**​ | **✔**​ | **✔** |
| Bevorder inspanningen om gemarginaliseerde ECRs te ondersteunen* | Bevorder een cultuur van diversiteit en inclusie | **-** | **✔**​ | **✔**​ | **✔**​ | **✔**​ | **✔**​ | **✔**​ |
|  | Identificeer en verminder barrières voor volledige deelname | **$/-** | **✔**​ | **✔**​ | **✔**​ | **✔**​ | **✔**​ | **✔**​ |
|  | Stel een beleid vast om ervoor te zorgen dat gemarginaliseerde groepen worden vertegenwoordigd in leidinggevende posities. | **$/-** | **✔**​ | **✔**​ | **✔**​ | **✔**​ | **✔**​ | **​A** |
| Steun globale initiatieven die de wetenschapscultuur en -praktijk verbeteren | Houd virtuele of hybride conferenties en netwerkevenementen, of gebruik een format die asynchrone deelname mogelijk maken (bijvoorbeeld door virtueel brainstormen). | **$/-** | ​ | **✔**​ | **✔**​ | **✔**​ | **✔**​ | **​A** |
|  | Bied subsidies voor activiteiten die de wetenschap verbeteren voor ECRs in landen of gemeenschappen met beperkte onderzoeksfinanciering | **$** | ​ | **✔**​ | ​ | **✔** | ​ | **​A** |
|  | Wetenschappers uit landen waar onderzoek relatief goed wordt gefinancierd, moeten kansen identificeren om de inspanningen van mensen met beperktere middelen te vergroten. | **$/-** | **✔**​ | **✔**​ | **✔**​ | **✔**​ | **✔**​ | **✔**​ |
|  | Neem bij het toevoegen van ECR-vertegenwoordigers aan commissies ECRs op uit landen met beperkte onderzoeksfinanciering en zorg ervoor dat deze diversiteit ook tot uiting komt bij niet-ECR commissieleden. | **$/-** | ​ | ​ | **✔**​ | **✔**​ | **✔**​ | **​A** |

***Tabel S3. Acties die organisaties en individuen kunnen nemen om ECRs te ondersteunen bij het verbeteren van wetenschapspublicatie en de wetenschapscultuur***

*Vinkjes geven specifieke acties aan die individuen of organisaties kunnen nemen voor het stimuleren en ondersteunen van ECR-activiteiten om de wetenschap te verbeteren.*

*De letter A duidt op acties waar collega’s, leidinggevenden of begeleiders voor kunnen pleiten door gebruik te maken van functies die zij binnen een organisatie bekleden.*

** Individuen en organisaties dienen de drie onderstaande aanbevelingen over te nemen bij alle wetenschappelijke inspanningen, inclusief hun wetenschappelijk werk en bij het uitvoeren van acties die in deze tabel worden beschreven. Raadpleeg de huidige bronnen voor de beste werkwijzen, aangezien praktijken op het gebied van diversiteit, gelijkheid en inclusie contextafhankelijk zijn en over tijd veranderen.*
