# Supplementary material for: Recommendations for empowering early career researchers to improve research culture and practice
Source: PLoS Biol. 2022 Jul 7;20(7):e3001680. doi: 10.1371/journal.pbio.3001680 (PMC9295962; doi:10.1371/journal.pbio.3001680)
Supplement: S4 Table — Maßnahmen, die Organisationen und Einzelpersonen ergreifen können, um ECRs bei der Verbesserung von wissenschaftlichen Veröffentlichungen und der Forschungskultur zu unterstützen. Häkchen kennzeichnen spezifische Maßnahmen, die Einzelpersonen oder Organisationen ergreifen können, um ECR-geleitete-Aktivitäten zur Verbesserung der Wissenschaft zu unterstützen und zu verstärken. Der Buchstabe A kennzeichnet Maßnahmen, für die sich Verbündete, Vorgesetzte oder Mentoren im Rahmen ihrer Positionen innerhalb einer Organisation einsetzen können. * Einzelpersonen und Organisationen sollten die drei folgenden Empfehlungen bei allen wissenschaftlichen Bemühungen einschließlich ihrer wissenschaftlichen Arbeit und bei der Umsetzung der in dieser Tabelle beschriebenen Maßnahmen berücksichtigen. Konsultieren Sie aktuelle Best-Practice-Ressourcen, da Praktiken zur Förderung von Vielfalt, Gleichberechtigung und Integration kontextabhängig sind und sich mit der Zeit weiterentwickeln. (DOCX) [file pbio.3001680.s013.docx]

**Empfehlungen für die Förderung von Nachwuchswissenschaftler:innen zur Verbesserung der Forschungskultur und -praxis**

| **Empfehlung** | **Unterstützende Maßnahmen** | **Kosten** | **Einrichtungen und Abteilungen** | **Fördermittelgeber** | **Journals und Verlage** | **Wissenschaftliche Fachgesellschaften** | **ECR Peer Gemeinschaften** | **Verbündete, Vorgesetzte und Mentoren** |
| --- | --- | --- | --- | --- | --- | --- | --- | --- |
| Schaffung von Aufstiegsmöglichkeiten durch Belohnungen und Anreize für Maßnahmen zur Verbesserung der Wissenschaft | Schaffung von Stellen für Meta-Forscher:innen und andere, die an der Verbesserung der Wissenschaft arbeiten | **$** | **✔** | **✔** | **✔** | **✔** |  | **A** |
|  | Belohnung von Aktivitäten zur Verbesserung der Wissenschaft bei Einstellung und Beförderung | **-** | **✔** | **✔** | **✔** | **✔** |  | **A** |
|  | Einbezug von Aktivitäten zur Verbesserung der Wissenschaft in die Bewertung von Ausbildungsstipendien | **-** | **✔** | **✔** |  |  |  | **A** |
|  | Publizieren von Meta-Forschung und Veröffentlichungen zur Verbesserung der Wissenschaft (idealerweise mit Open Access) | **$/-** |  |  | **✔** |  |  | **A** |
|  | Anbieten von Auszeichnungen für Aktivitäten zur Verbesserung der Wissenschaft | **$/-** | **✔** | **✔** | **✔** | **✔** | **✔** | **A** |
| Integration von ECRs in Entscheidungsprozesse | Einrichtung von Beratungsgruppen, die sich aus ECRs zusammensetzen, und Aufrechterhaltung eines intensiven Dialogs mit Entscheidungsgremien | **$/-** | **✔** | **✔** | **✔** | **✔** |  | **A** |
|  | Einbezug von ECR-Vertreter:innen in wissenschaftliche Ausschüsse; Schaffung einer einladenden und unterstützenden Atmosphäre | **$/-** | **✔** | **✔** | **✔** | **✔** |  | **A** |
|  | Erwägung der Kombination von ECR-Beratungsgruppen mit ECR-Vertreter:innen in Ausschüssen | **$/-** | **✔** | **✔** | **✔** | **✔** |  | **A** |
| Bereitstellung von Ressourcen, Finanzmitteln und geschützter Zeit für ECRs, die sich mit der Verbesserung der Forschung befassen | Schaffung von Stipendien zur Verbesserung der Wissenschaft; Sicherstellung, dass ECRs antragsberechtigt sind | **$** | **✔** | **✔** | **✔** | **✔** |  | **A** |
|  | Einrichtung kleiner Stipendien für ECRs, die Ideen zur Verbesserung des wissenschaftlichen Publikationswesens haben | **$** |  | **✔** | **✔** | **✔** |  | **A** |
|  | Anbieten von logistischer oder administrativer Unterstützung für ECR-Initiativen (z.B. in Form eines Community Managers) | **$** | **✔** | **✔** | **✔** | **✔** |  | **A** |
|  | Veröffentlichung von Programmen oder Ergebnissen, die für die ECR-Gemeinschaft von Nutzen sind | **$/-** | **✔** | **✔** | **✔** | **✔** | **✔** | **✔** |
|  | Anbieten von Stipendien, die ECRs geschützte Zeit für Aktivitäten zur Verbesserung der Forschung bieten | **$** | **✔** | **✔** |  | **✔** |  | **A** |
|  | Ermutigung der ECRs, Aktivitäten zur Verbesserung der Wissenschaft in ihre Karrierepläne einzubeziehen | **-** | **✔** | **✔** |  | **✔** |  | **✔** |
| Anerkennung des Fachwissens von ECRs und Verstärkung ihrer Bemühungen zur Verbesserung der Wissenschaft | Schaffung von (Online-)Gemeinschaften für ECRs, die sich für die Verbesserung der Wissenschaftskultur und -praxis einsetzen | **$/-** | **✔** | **✔** | **✔** | **✔** | **✔** | **A** |
|  | Ausbildung von Wissenschaftler:innen in Fähigkeiten, die zur Verbesserung der Wissenschaft auf individueller und systemischer Ebene erforderlich sind | **$/-** | **✔** | **✔** | **✔** | **✔** | **✔** | **A** |
|  | Ehrliches, konstruktives Feedback geben, um ECRs bei der Fehlersuche und der Verfeinerung von Ideen zu helfen | **-** | **✔** | **✔** | **✔** | **✔** | **✔** | **✔** |
|  | Nutzung von Maßnahmen zur Verbesserung bestehender Forschungsprojekte | **$/-** | **✔** | **✔** | **✔** | **✔** | **✔** | **✔** |
|  | Zusammenarbeit mit den ECRs, um sicherzustellen, dass die Verbesserungen auch nach dem Ausscheiden der ECRs Bestand haben, indem Änderungen in Standardarbeitsanweisungen oder Laborhandbücher aufgenommen werden | **-** | **✔** | **✔** | **✔** | **✔** | **✔** | **✔** |
|  | Erhöhung der Sichtbarkeit von ECR-geführten Bemühungen zur Verbesserung der Wissenschaft; ECRs sollen die Möglichkeit erhalten, ihre Aktivitäten zur Verbesserung der Forschung mit anderen zu teilen | **$/-** | **✔** | **✔** | **✔** | **✔** | **✔** | **✔** |
| Förderung von Bemühungen zur Unterstützung marginalisierter ECRs* | Förderung einer Kultur der Vielfalt und Integration | **-** | **✔** | **✔** | **✔** | **✔** | **✔** | **✔** |
|  | Identifikation und Beseitigung von Hindernissen für eine umfassendere Beteiligung | **$/-** | **✔** | **✔** | **✔** | **✔** | **✔** | **✔** |
|  | Maßnahmen ergreifen, um die Vertretung von marginalisierten Gruppen in Führungspositionen zu gewährleisten | **$/-** | **✔** | **✔** | **✔** | **✔** | **✔** | **A** |
| Unterstützung globaler Initiativen zur Verbesserung der Forschungskultur und -praxis | Veranstalten von virtuellen oder hybriden Konferenzen und Netzwerkveranstaltungen oder Nutzen von Formaten, die eine asynchrone Teilnahme ermöglichen (z. B. virtuelles Brainstorming) | **$/-** |  | **✔** | **✔** | **✔** | **✔** | **A** |
|  | Bereitstellung von Zuschüssen zur Verbesserung der Forschung von ECRs in Ländern oder Gemeinschaften mit begrenzten Forschungsmitteln | **$** |  | **✔** |  | **✔** |  | **A** |
|  | Wissenschaftler:innen aus Ländern, in denen die Forschung vergleichsweise gut finanziert ist, sollten nach Möglichkeiten suchen, die Anstrengungen derjenigen zu verstärken, die über weniger Mittel verfügen | **$/-** | **✔** | **✔** | **✔** | **✔** | **✔** | **✔** |
|  | Bei der Aufnahme von ECR-Vertreter:innen in Ausschüsse sollten auch ECRs aus Ländern mit begrenzter Forschungsfinanzierung einbezogen werden. Es sollte sichergestellt sein, dass sich diese Vielfalt auch unter den Nicht-ECR-Ausschussmitgliedern widerspiegelt | **$/-** |  |  | **✔** | **✔** | **✔** | **A** |

***Tabelle S4.*** ***Maßnahmen, die Organisationen und Einzelpersonen ergreifen können, um ECRs bei der Verbesserung von wissenschaftlichen Veröffentlichungen und der Forschungskultur zu unterstützen***

*Häkchen kennzeichnen spezifische Maßnahmen, die Einzelpersonen oder Organisationen ergreifen können, um ECR-geleitete-Aktivitäten zur Verbesserung der Wissenschaft zu unterstützen und zu verstärken. Der Buchstabe A kennzeichnet Maßnahmen, für die sich Verbündete, Vorgesetzte oder Mentoren im Rahmen ihrer Positionen innerhalb einer Organisation einsetzen können.*

** Einzelpersonen und Organisationen sollten die drei folgenden Empfehlungen bei allen wissenschaftlichen Bemühungen einschließlich ihrer wissenschaftlichen Arbeit und bei der Umsetzung der in dieser Tabelle beschriebenen Maßnahmen berücksichtigen. Konsultieren Sie aktuelle Best-Practice-Ressourcen, da Praktiken zur Förderung von Vielfalt, Gleichberechtigung und Integration kontextabhängig sind und sich mit der Zeit weiterentwickeln.*
