# Supplementary material for: Recommendations for empowering early career researchers to improve research culture and practice
Source: PLoS Biol. 2022 Jul 7;20(7):e3001680. doi: 10.1371/journal.pbio.3001680 (PMC9295962; doi:10.1371/journal.pbio.3001680)
Supplement: S5 Table — Azioni che le organizzazioni e gli individui posso adottare per supportare i ricercatori nelle fasi iniziali della loro carriera nel miglioramento dell’editoria scientifica e della cultura della ricerca. I simboli di spunta (✔) indicano specifiche azioni che gli individui o le organizzazioni posso adottare per supportare ed amplificare le attività proposte dai giovani ricercatori per migliorare la scienza. La lettera “A” denota le azioni che alleati, supervisori e mentori posso perseguire data la loro posizione all’interno delle organizzazioni di cui fanno parte. *Gli individui e le organizzazioni dovrebbero adottare queste tre raccomandazioni in tutti gli sforzi di ricerca, inclusi il loro lavoro scientifico e quando stiano implementando ognuna delle azioni descritte in questa tabella. Si consultino le risorse riguardo le buone prassi correnti in quanto le pratiche riguardo la diversità, l’equità e l’inclusione dipendono dai contesti ed evolvono nel corso del tempo (DOCX) [file pbio.3001680.s014.docx]

**Raccomandazioni per consentire ai ricercatori nelle fasi iniziali della loro carriera di migliorare la cultura e le pratiche nella ricerca**

| **Raccomandazione**​ | **Azioni a support** | **Costo** | **Istituzioni e Dipartimenti** | **Agenzie di Finanziamento** | **Riviste ed Editori** | **Società scientifiche**​ | **Comunità di giovani ricercatori**​ | **Alleati, Supervisori e Mentori**​ |
| --- | --- | --- | --- | --- | --- | --- | --- | --- |
| Fornire un percorso per gli avanzamenti di carriera incentivando e premiando le attività di miglioramento della scienza​ | Creare delle posizioni per meta-ricercatori e altri ruoli lavorativi focalizzati sul miglioramento della scienza | **$** | **✔**​ | **✔**​ | **✔**​ | **✔**​ | ​ | **​A** |
|  | Premiare le attività di miglioramento della scienza per assunzioni e promozioni lavorative | **-** | **✔**​ | **✔**​ | **✔**​ | **✔**​ | ​ | **​A** |
|  | Includere le attività di miglioramento della scienza nelle valutazioni dei finanziamenti di attività didattiche | **-** | **✔**​ | **✔**​ | ​ | ​ | ​ | **​A** |
|  | Pubblicare delle meta-ricerche e articoli sul miglioramento della scienza (idealmente ad accesso aperto) | **$/-** | ​ | ​ | **✔**​ | ​ | ​ | **​A** |
|  | Offrire dei premi per le attività di miglioramento della scienza | **$/-** | **✔**​ | **✔**​ | **✔**​ | **✔**​ | **✔** | **​A** |
| Integrare i giovani ricercatori nei processi decisionali | Creare dei gruppi di consulenza composti da giovani ricercatore e mantenere un forte dialogo con le entità decisionali ​ | **$/-** | **✔**​ | **✔**​ | **✔**​ | **✔**​ | ​ | **​A** |
|  | Includere dei rappresentati dei giovani ricercatori nei comitati scientifici, creando un’atmosfera accogliente e di supporto | **$/-** | **✔**​ | **✔**​ | **✔**​ | **✔**​ | ​ | **​A** |
|  | Considerare l’inclusione nei comitati di giovani ricercatori facenti parte di gruppi di consulenza con rappresentati dei giovani ricercatori | **$/-** | **✔**​ | **✔**​ | **✔**​ | **✔**​ | ​ | **​A** |
| Procurare risorse, finanziamenti, tempo riservato ai giovani ricercatori che sono dediti al miglioramento della ricerca affinché’ possano dedicarsi a queste attività e al cambiamento delle buone prassi. | Creare dei finanziamenti per il miglioramento della scienza; assicurarsi che i giovani ricercatori siano ammessi ai bandi | **$** | **✔**​ | **✔**​ | **✔**​ | **✔**​ | ​ | **​A** |
|  | Creare dei piccoli finanziamenti per i giovani ricercatori che hanno idee su come migliorare l’editoria scientifica​ | **$** | ​ | **✔**​ | **✔**​ | **✔**​ | ​ | **​A** |
|  | Offrire supporto logistico ed amministrativo alle iniziative per giovani ricercatori (ad esempio un manager della comunità) | **$** | **✔**​ | **✔**​ | **✔**​ | **✔**​ | ​ | **​A** |
|  | Pubblicizzare i programmi e prodotti rilevanti per la comunità dei giovani ricercatori | **$/-** | **✔**​ | **✔**​ | **✔**​ | **✔**​ | **✔** | **✔**​ |
|  | Offrire dei finanziamenti che forniscano ai giovani ricercatori del tempo riservato ad attività di miglioramento della ricerca | **$** | **✔**​ | **✔**​ | ​ | **✔**​ | ​ | **​A** |
|  | Incoraggiare i giovani ricercatori ad includere attività per il miglioramento della scienza nei loro programmi di avanzamento di carriera | **-** | **✔**​ | **✔**​ | ​ | **✔**​ | ​ | **✔**​ |
| Riconoscere l’esperienza dei giovani ricercatore ed amplificare i loro sforzi per il miglioramento della scienza ​  ​  ​ | Creare comunità (eventualmente virtuali) dove i giovani ricercatori possano lavorare al miglioramento della cultura e prassi scientifiche​ | **$/-** | **✔**​ | **✔**​ | **✔**​ | **✔**​ | **✔**​ | **​A** |
|  | Insegnare agli scienziati le competenze necessarie per migliorare la scienza a livello personale e di sistema | **$/-** | **✔**​ | **✔**​ | **✔**​ | **✔**​ | **✔**​ | **​A** |
|  | Fornire un riscontro onesto e costruttivo per aiutare i giovani ricercatori a correggere e rifinire le loro idee | **-** | **✔**​ | **✔**​ | **✔**​ | **✔**​ | **✔**​ | **✔**​ |
|  | Usare le attività di miglioramento della ricerca per potenziare i progetti esistenti ​ | **$/-** | **✔**​ | **✔**​ | **✔**​ | **✔**​ | **✔**​ | **✔**​ |
|  | Lavorare con i giovani ricercatori per garantire che i miglioramenti siano sostenibili anche una volta terminato il lavoro del ricercatore, integrando i cambiamenti nelle procedure standard o nei manuali di laboratorio | **-** | **✔**​ | **✔**​ | **✔**​ | **✔**​ | **✔**​ | **✔**​ |
|  | Aumentare la visibilità degli sforzi portati avanti dai giovani ricercatori nello sviluppo della scienza, dare opportunità ai giovani ricercatori di condividere i loro progressi con altri | **$/-** | **✔**​ | **✔**​ | **✔**​ | **✔**​ | **✔**​ | **✔** |
| Sostenere gli sforzi a supporto dei giovani ricercatori marginalizzati​* | Incoraggiare una cultura di diversità ed inclusione ​ | **-** | **✔**​ | **✔**​ | **✔**​ | **✔**​ | **✔**​ | **✔**​ |
|  | Identificare ed eliminare le barriere ad una piena partecipazione | **$/-** | **✔**​ | **✔**​ | **✔**​ | **✔**​ | **✔**​ | **✔**​ |
|  | Attivare delle policy che assicurino ai gruppi marginalizzati una adeguata rappresentanza in posizioni comando | **$/-** | **✔**​ | **✔**​ | **✔**​ | **✔**​ | **✔**​ | **​A** |
| Supportare le iniziative globali per il miglioramento della cultura e pratiche di ricerca | Ospitare delle conferenze ed eventi di networking virtuali o ibridi, o usare formati che rendano possibile la partecipazione asincrona degli invitati (ad esempio dei brainstorming virtuali) | **$/-** | ​ | **✔**​ | **✔**​ | **✔**​ | **✔**​ | **​A** |
|  | Offrire dei finanziamenti per il miglioramento della ricercar per i giovani ricercatori in nazioni o comunità con un limitato accesso ai finanziamenti | **$** | ​ | **✔**​ | ​ | **✔** | ​ | **​A** |
|  | Gli scienziati provenienti da nazioni dove la ricerca è relativamente ben finanziata dovrebbero identificare delle opportunità per amplificare gli sforzi dei colleghi con meno risorse | **$/-** | **✔**​ | **✔**​ | **✔**​ | **✔**​ | **✔**​ | **✔**​ |
|  | Quando si aggiungono i rappresentanti dei giovani ricercatori nei comitati, includere giovani ricercatori provenienti da nazioni con limitate risorse dedicate alla ricerca. Questa diversità andrebbe ricercata anche nei membri di comitato in stadi più avanzati della loro carriera | **$/-** | ​ | ​ | **✔**​ | **✔**​ | **✔**​ | **​A** |

***Tabella S5.*** ***Azioni che le organizzazioni e gli individui posso adottare per supportare i ricercatori nelle fasi iniziali della loro carriera nel miglioramento dell’editoria scientifica e della cultura della ricerca.***

*I simboli di spunta (✔) indicano specifiche azioni che gli individui o le organizzazioni posso adottare per supportare ed amplificare le attività proposte dai giovani ricercatori per migliorare la scienza. La lettera “A” denota le azioni che alleati, supervisori e mentori posso perseguire data la loro posizione all’interno delle organizzazioni di cui fanno parte. *Gli individui e le organizzazioni dovrebbero adottare queste tre raccomandazioni in tutti gli sforzi di ricerca, inclusi il loro lavoro scientifico e quando stiano implementando ognuna delle azioni descritte in questa tabella. Si consultino le risorse riguardo le buone prassi correnti in quanto le pratiche riguardo la diversità, l’equità e l’inclusione dipendono dai contesti ed evolvono nel corso del tempo*
