# Supplementary material for: Recommendations for empowering early career researchers to improve research culture and practice
Source: PLoS Biol. 2022 Jul 7;20(7):e3001680. doi: 10.1371/journal.pbio.3001680 (PMC9295962; doi:10.1371/journal.pbio.3001680)
Supplement: S6 Table — 科学出版と研究文化の改革に向けてECRを支援するために組織と個人が取るべき行動.チェックマークは、科学を向上させるためのECR活動を支援・拡大するために、個人または組織が取ることのできる具体的な行動を示す。Aは指導教員、メンターが組織内の役職の一部として提唱できる行動を示す。* 科学的活動を含むすべての活動において、この表に記載された行動を実施する際に、個人および組織は以下の3つの提言を採用すべきである。多様性、公平性、包括性の実践は状況に依存し、時とともに変化するため、現行の最良事例を参照する。 (DOCX) [file pbio.3001680.s015.docx]

**研究の文化と実践をより良くするための、早期キャリア研究者強化に関する提言**

| **提言** | **支援活動** | **コスト** | **機関・専攻** | **資金提供機関** | **雑誌・出版社** | **科学協会** | **ECR の同僚コミュニティ​** | **同盟、指導教員・メンター** |
| --- | --- | --- | --- | --- | --- | --- | --- | --- |
| 科学の改革活動への報奨とインセンティブによるキャリアアップの機会を提供する | メタ研究者、その他科学の改革に取り組む人々のためのポジションを創設する | **$** | **✔**​ | **✔**​ | **✔**​ | **✔**​ | ​ | **​A** |
|  | 採用・昇進における科学の改革活動への評価 | **-** | **✔**​ | **✔**​ | **✔**​ | **✔**​ | ​ | **​A** |
|  | 科学の改革活動を研究助成の評価に組み入れる | **-** | **✔**​ | **✔**​ | ​ | ​ | ​ | **​A** |
|  | メタ研究および科学の改革に関する論文の出版（理想的にはオープンアクセス） | **$/-** | ​ | ​ | **✔**​ | ​ | ​ | **​A** |
|  | 科学の改革活動に対する表彰 | **$/-** | **✔**​ | **✔**​ | **✔**​ | **✔**​ | **✔** | **​A** |
| ECRを意思決定プロセスに組み込む | ECRで構成されるアドバイザリーグループを設立し、意思決定機関と密接な対話を維持する | **$/-** | **✔**​ | **✔**​ | **✔**​ | **✔**​ | ​ | **​A** |
|  | 科学委員会にECRの代表を参加させ、歓迎と協力の雰囲気を作る | **$/-** | **✔**​ | **✔**​ | **✔**​ | **✔**​ | ​ | **​A** |
|  | ECRアドバイザリーグループと委員会におけるECR代表の組み合わせの検討 | **$/-** | **✔**​ | **✔**​ | **✔**​ | **✔**​ | ​ | **​A** |
| 科学の改革に長けたECRに、研究文化と実践を改革するためのリソース、資金、保証された時間を提供する | 科学改革への助成金を創設し、ECRに申請資格があることを保証する | **$** | **✔**​ | **✔**​ | **✔**​ | **✔**​ | ​ | **​A** |
|  | 科学出版の改革に関するアイデアをもつECRに小さな助成金を創設する | **$** | ​ | **✔**​ | **✔**​ | **✔**​ | ​ | **​A** |
|  | ECRの取り組みに対する物流的または管理的支援の提供（例：コミュニティマネージャー） | **$** | **✔**​ | **✔**​ | **✔**​ | **✔**​ | ​ | **​A** |
|  | ECRコミュニティにとって価値あるプログラムまたは成果を公表する | **$/-** | **✔**​ | **✔**​ | **✔**​ | **✔**​ | **✔** | **✔**​ |
|  | ECRに研究の改革活動のための保護された時間を提供する助成金を提供する | **$** | **✔**​ | **✔**​ | ​ | **✔**​ | ​ | **​A** |
|  | ECRが科学の改革活動をキャリア形成計画に取り入れるよう促す | **-** | **✔**​ | **✔**​ | ​ | **✔**​ | ​ | **✔**​ |
| ECRの専門性を認識し、科学改革に向けた彼らの努力を増幅させる​  ​ | 研究の文化と実践の改革に取り組むECRのための（オンライン）コミュニティの創設 | **$/-** | **✔**​ | **✔**​ | **✔**​ | **✔**​ | **✔**​ | **​A** |
|  | 個人および組織レベルで科学を改革するために必要なスキルを持つ科学者を育成する | **$/-** | **✔**​ | **✔**​ | **✔**​ | **✔**​ | **✔**​ | **​A** |
|  | ECRのトラブルシューティングとアイデアの改良を支援するために、率直で建設的なフィードバックを提供する | **-** | **✔**​ | **✔**​ | **✔**​ | **✔**​ | **✔**​ | **✔**​ |
|  | 研究改革活動を通じて、既存のプロジェクトを強化する | **$/-** | **✔**​ | **✔**​ | **✔**​ | **✔**​ | **✔**​ | **✔**​ |
|  | ECRと協力して、ECRが異動した後も改革が持続するようにする。 変更を標準業務手順書や研究室マニュアルに反映させる。 | **-** | **✔**​ | **✔**​ | **✔**​ | **✔**​ | **✔**​ | **✔**​ |
|  | ECR主導の研究改革活動の認知度を高め、ECRが研究改革活動を他者と共有する機会を提供する | **$/-** | **✔**​ | **✔**​ | **✔**​ | **✔**​ | **✔**​ | **✔** |
| 社会から疎外されたECRを支援する取り組みを推進する* | 多様性と包括性の文化を育む | **-** | **✔**​ | **✔**​ | **✔**​ | **✔**​ | **✔**​ | **✔**​ |
|  | 完全な参加を阻む障壁の特定と排除 | **$/-** | **✔**​ | **✔**​ | **✔**​ | **✔**​ | **✔**​ | **✔**​ |
|  | 指導的立場にある社会から疎外された人々の代表を確保するための方針を制定する | **$/-** | **✔**​ | **✔**​ | **✔**​ | **✔**​ | **✔**​ | **​A** |
| 研究文化・実践の向上に向けたグローバルな取り組みを支援​ | バーチャルまたはハイブリッドな 会議や ネットワーキング イベントを主催する、あるいは非同期参加可能な形式を用いる（例：バーチャルブレインストーミング） | **$/-** | ​ | **✔**​ | **✔**​ | **✔**​ | **✔**​ | **​A** |
|  | ​研究資金が限られている国や地域のECRに対して、研究改革助成金を提供する | **$** | ​ | **✔**​ | ​ | **✔** | ​ | **​A** |
|  | 研究資金が比較的豊富な国の科学者は、資源の乏しい国の研究者の努力を増幅する機会を見出す必要がある | **$/-** | **✔**​ | **✔**​ | **✔**​ | **✔**​ | **✔**​ | **✔**​ |
|  | 委員会にECRの代表を加える場合は、研究資金が限られている国のECRを含める。この多様性は、ECR以外の委員会メンバーにも適用されるようにする​ | **$/-** | ​ | ​ | **✔**​ | **✔**​ | **✔**​ | **​A** |

***Table S6.*** ***科学出版と研究文化の改革に向けてECRを支援するために組織と個人が取るべき行動***

*チェックマークは、科学を向上させるためのECR活動を支援・拡大するために、個人または組織が取ることのできる具体的な行動を示す。Aは指導教員、メンターが組織内の役職の一部として提唱できる行動を示す。* 科学的活動を含むすべての活動において、この表に記載された行動を実施する際に、個人および組織は以下の3つの提言を採用すべきである。多様性、公平性、包括性の実践は状況に依存し、時とともに変化するため、現行の最良事例を参照する。*
