# Supplementary material for: Recommendations for empowering early career researchers to improve research culture and practice
Source: PLoS Biol. 2022 Jul 7;20(7):e3001680. doi: 10.1371/journal.pbio.3001680 (PMC9295962; doi:10.1371/journal.pbio.3001680)
Supplement: S7 Table — Działania, które organizacje i osoby fizyczne mogą podjąć w celu wsparcia ECR w doskonaleniu publikacji naukowych i kultury badawczej. Zaznaczone pola wskazują konkretne działania, które osoby lub organizacje mogą podjąć w celu wsparcia i wzmocnienia działań ECR w celu poprawy nauki. Litera A oznacza działania, które promotorzy, przełożeni lub mentorzy mogą popierać w ramach stanowisk, które zajmują w organizacji. * Osoby i organizacje powinny przyjąć trzy poniższe zalecenia we wszystkich przedsięwzięciach naukowych, w tym w swojej pracy naukowej i podczas wdrażania wszelkich działań opisanych w tej tabeli. Zapoznaj się z aktualnymi zasobami najlepszych praktyk, ponieważ praktyki w zakresie różnorodności, równości i integracji zależą od kontekstu i ewoluują z czasem. (DOCX) [file pbio.3001680.s016.docx]

**Zalecenia dotyczące wzmocnienia pozycji początkujących naukowców w celu poprawy kultury i praktyki badawczej**

| **Zalecenia** | **Działania wspierające** | **Koszt** | **Instytuty i wydziały** | **Agencje finansujące** | **Czasopisma i wydawnictwa** | **Organizacje naukowe** | **Społeczności początkujących naukowców** | **Promotorzy, przełożeni  i mentorzy** |
| --- | --- | --- | --- | --- | --- | --- | --- | --- |
| Zapewniać  ścieżkę rozwoju kariery, nagradzając i zachęcając do działań na rzecz doskonalenia nauki | Tworzenie stanowisk dla meta-badaczy oraz osób pracujących na rzecz poprawy jakości badań | **$** | **✔** | **✔** | **✔** | **✔** |  | **A** |
|  | Nagradzanie działalności na rzecz  poprawy jakości badań naukowych poprzez awans zawodowy | **-** | **✔** | **✔** | **✔** | **✔** |  | **A** |
|  | Włączanie oceny działań na rzecz poprawy jakości badań do szkoleń oceniania grantów | **-** | **✔** | **✔** |  |  |  | **A** |
|  | Publikowanie prac na temat meta-nauki oraz poprawy jakości badań (najlepiej w formacie open access) | **$/-** |  |  | **✔** |  |  | **A** |
|  | Oferowanie nagród za działania mające na celu doskonalenie  nauki | **$/-** | **✔** | **✔** | **✔** | **✔** | **✔** | **A** |
| Uwzględniać początkujących naukowców  w procesach decyzyjnych | Tworzenie grup doradczych złożonych z początkujących naukowców i podtrzymywanie dialogu z organami decyzyjnymi | **$/-** | **✔** | **✔** | **✔** | **✔** |  | **A** |
|  | Włączanie przedstawicieli początkujących naukowców do komitetów naukowych, tworzenie przyjaznej i wspierającej atmosfery | **$/-** | **✔** | **✔** | **✔** | **✔** |  | **A** |
|  | Rozważenie połączenia grup doradczych  początkujących naukowców z przedstawicielami początkujących naukowców  w komisjach | **$/-** | **✔** | **✔** | **✔** | **✔** |  | **A** |
| Zapewniać  zasoby, finansowanie oraz chroniony czas potrzebny na poprawę badań dla ECR, którzy są wykwalifikowani w doskonaleniu badań​ | Tworzenie grantów na poprawę stanu nauki; zapewnienie warunków, w których początkujący naukowcy kwalifikują się do takowych grantów | **$** | **✔** | **✔** | **✔** | **✔** |  | **A** |
|  | Tworzenie małych grantów dla młodych naukowców, którzy mają pomysły na poprawę jakości publikacji naukowych | **$** |  | **✔** | **✔** | **✔** |  | **A** |
|  | Oferowanie wsparcia logistycznego i administracyjnego dla inicjatyw młodych naukowców (np. Manadżer społecznościowy) | **$** | **✔** | **✔** | **✔** | **✔** |  | **A** |
|  | Publikowanie wyników oraz programów wartościowych dla ECR | **$/-** | **✔** | **✔** | **✔** | **✔** | **✔** | **✔** |
|  | Oferowanie grantów, które oferują ECR chroniony czas na działania doskonalące badania | **$** | **✔** | **✔** |  | **✔** |  | **A** |
|  | Zachęcanie ECR do włączania działań na rzecz doskonalenia nauki do planów rozwoju kariery | **-** | **✔** | **✔** |  | **✔** |  | **✔** |
| Uznać wiedzę ekspercką początkujących naukowców  i zwiększenie ich wysiłków na rzecz doskonalenia nauki | Tworzenie (internetowych) społeczności dla ECR pracujących nad poprawą kultury i praktyk naukowych | **$/-** | **✔** | **✔** | **✔** | **✔** | **✔** | **A** |
|  | Szkolenie naukowców w zakresie umiejętności potrzebnych do poprawy nauki na poziomie indywidualnym i systemowym | **$/-** | **✔** | **✔** | **✔** | **✔** | **✔** | **A** |
|  | Przekazywanie  szczerej, konstruktywnej opinii, aby pomóc ECR w rozwiązywaniu problemów i udoskonalaniu pomysłów | **-** | **✔** | **✔** | **✔** | **✔** | **✔** | **✔** |
|  | Wykorzystywanie  działań  w zakresie doskonalenia badań, aby ulepszyć istniejące projekty | **$/-** | **✔** | **✔** | **✔** | **✔** | **✔** | **✔** |
|  | Współpraca z ECR w celu zapewnienia, że ulepszenia są trwałe poprzez integrację zmian w standardowych procedurach operacyjnych lub podręcznikach laboratoryjnych | **-** | **✔** | **✔** | **✔** | **✔** | **✔** | **✔** |
|  | Zwiększenie widoczności wysiłków podejmowanych przez ECR w celu poprawy stanu nauki; dawanie ECR możliwości dzielenia się swoimi działaniami w zakresie doskonalenia badań z innymi | **$/-** | **✔** | **✔** | **✔** | **✔** | **✔** | **✔** |
| Wspierać marginalizowanych ECR* | Wspieranie kultury różnorodności | **-** | **✔** | **✔** | **✔** | **✔** | **✔** | **✔** |
|  | Identyfikacja i eliminacja barier na drodze do pełnego uczestnictwa | **$/-** | **✔** | **✔** | **✔** | **✔** | **✔** | **✔** |
|  | Uchwalenie polityk zapewniających reprezentację marginalizowanych grup na stanowiskach kierowniczych | **$/-** | **✔** | **✔** | **✔** | **✔** | **✔** | **A** |
| Wspierać globalne inicjatywy na rzecz poprawy kultury i praktyk badawczych | Organizowanie wirtualnych lub hybrydowych konferencji i wydarzeń  lub używanie  formatów umożliwiających asynchroniczne uczestnictwo (np. wirtualna burza mózgów) | **$/-** |  | **✔** | **✔** | **✔** | **✔** | **A** |
|  | Oferowanie grantów  na poprawę badań dla ECR w krajach lub społecznościach o ograniczonym finansowaniu badań | **$** |  | **✔** |  | **✔** |  | **A** |
|  | Naukowcy z krajów, w których badania są stosunkowo dobrze finansowane, powinni zidentyfikować możliwości zwiększenia wysiłków osób o mniejszych zasobach. | **$/-** | **✔** | **✔** | **✔** | **✔** | **✔** | **✔** |
|  | Dodawanie  przedstawicieli ECR do komitetów, uwzględniając  ECR z krajów o ograniczonym finansowaniu badań. Zapewnienie, aby ta różnorodność znalazła odzwierciedlenie również wśród członków komisji spoza ECR. | **$/-** |  |  | **✔** | **✔** | **✔** | **A** |

**Tabela S7. Działania, które organizacje i osoby fizyczne mogą podjąć w celu wsparcia ECR w doskonaleniu publikacji naukowych i kultury badawczej**

*Zaznaczone pola  wskazują konkretne działania, które osoby lub organizacje mogą podjąć w celu wsparcia i wzmocnienia działań ECR w celu poprawy nauki. Litera A oznacza działania, które promotorzy, przełożeni lub mentorzy mogą popierać w ramach stanowisk, które zajmują w organizacji. * Osoby i organizacje powinny przyjąć trzy poniższe zalecenia we wszystkich przedsięwzięciach naukowych, w tym w swojej pracy naukowej i podczas wdrażania wszelkich działań opisanych w tej tabeli. Zapoznaj się z aktualnymi zasobami najlepszych praktyk, ponieważ praktyki w zakresie różnorodności, równości i integracji zależą od kontekstu i ewoluują z czasem.*
