# Supplementary material for: Recommendations for empowering early career researchers to improve research culture and practice
Source: PLoS Biol. 2022 Jul 7;20(7):e3001680. doi: 10.1371/journal.pbio.3001680 (PMC9295962; doi:10.1371/journal.pbio.3001680)
Supplement: S8 Table — Ações que organizações e indivíduos podem adotar para apoiar os ECRs na melhoria da publicação científica e da cultura de investigação. As marcas de seleção indicam ações específicas que indivíduos ou organizações podem realizar para apoiar e amplificar as atividades de ECRs para melhorar a ciência. A letra A denota ações que aliados, supervisores ou mentores podem defender como parte dos cargos que ocupam dentro de uma organização. * Indivíduos e organizações devem adotar as três recomendações abaixo em todos os empreendimentos científicos, incluindo no seu trabalho científico e ao implementar quaisquer ações descritas nesta tabela. Consulte os recursos atuais de melhores práticas, pois as práticas de diversidade, equidade e inclusão dependem do contexto e evoluem ao longo do tempo. (DOCX) [file pbio.3001680.s017.docx]

**Recomendações para o Empoderamento de Investigadores em Início de Carreira para Melhorar a Cultura e a Prática Científica**

| **Recomendação** | **Ações de apoio** | **Custo** | **Instituições & Departmentos** | **Agências de financiamento** | **Jornais &**​**Editores** | **Sociedades científicas** | **Comunidades de ECR correspondentes**​ | **Aliados, Supervisores & Mentorse**​ |
| --- | --- | --- | --- | --- | --- | --- | --- | --- |
| Fornecer um caminho para a progressão de carreira, recompensando e incentivando atividades de melhoramento científico ​ | Criar posições para meta-investigadores & outros que trabalham para melhorar a ciência | **$** | **✔**​ | **✔**​ | **✔**​ | **✔**​ | ​ | **​A** |
|  | Recompensar atividades de melhoria científica no recrutamento & promoção | **-** | **✔**​ | **✔**​ | **✔**​ | **✔**​ | ​ | **​A** |
|  | Incorporar atividades de melhoria científica nas avaliações de bolsas de estágio | **-** | **✔**​ | **✔**​ | ​ | ​ | ​ | **​A** |
|  | Publicar artigos de meta-pesquisa & melhoramento científico (idealmente open access) | **$/-** | ​ | ​ | **✔**​ | ​ | ​ | **​A** |
|  | Oferecer prémios para atividades de melhoramento científico ​ | **$/-** | **✔**​ | **✔**​ | **✔**​ | **✔**​ | **✔** | **​A** |
| Integrar ECRs em processos de tomada de decisão | Criar grupos de aconselhamento compostos por ECRs & manter um forte diálogo com os órgãos de tomada de decisão | **$/-** | **✔**​ | **✔**​ | **✔**​ | **✔**​ | ​ | **​A** |
|  | Incluir ECR representantes em comités científicos; criar uma atmosfera acolhedora & de apoio | **$/-** | **✔**​ | **✔**​ | **✔**​ | **✔**​ | ​ | **​A** |
|  | Considere combinar grupos de aconselhamento de ECRs com representantes de ECR em comités | **$/-** | **✔**​ | **✔**​ | **✔**​ | **✔**​ | ​ | **​A** |
| Fornecer ECRs qualificados em melhoria de investigação com recursos, financiamento e tempo dedicado para melhorar a cultura & a prática de investigação | Criar subsídios de melhoramento científico; Certifique-se de que os ECRs são elegíveis para concorrer | **$** | **✔**​ | **✔**​ | **✔**​ | **✔**​ | ​ | **​A** |
|  | Crie pequenos subsídios para ECRs que tenham ideias sobre como melhorar a publicação científica | **$** | ​ | **✔**​ | **✔**​ | **✔**​ | ​ | **​A** |
|  | Oferecer apoio logístico ou administrativo para iniciativas de ECR (por exemplo, um gestor de comunidade) | **$** | **✔**​ | **✔**​ | **✔**​ | **✔**​ | ​ | **​A** |
|  | Divulgue programas ou resultados importantes para a comunidade de ECR | **$/-** | **✔**​ | **✔**​ | **✔**​ | **✔**​ | **✔** | **✔**​ |
|  | Oferecer subsídios que forneçam aos ECRs tempo dedicado para atividades de melhoria de investigação | **$** | **✔**​ | **✔**​ | ​ | **✔**​ | ​ | **​A** |
|  | Incentivar os ECRs a incorporar atividades de melhoria científica nos planos de desenvolvimento de carreira | **-** | **✔**​ | **✔**​ | ​ | **✔**​ | ​ | **✔**​ |
| Reconhecer a experiência dos ECRs e amplificar os seus esforços para melhorar a ciência  ​  ​ | Criar comunidades (online) para ECRs que trabalham para melhorar a cultura e as práticas científicas | **$/-** | **✔**​ | **✔**​ | **✔**​ | **✔**​ | **✔**​ | **​A** |
|  | Treinar cientistas nas qualificações necessárias para melhorar a ciência a nível individual & sistémico | **$/-** | **✔**​ | **✔**​ | **✔**​ | **✔**​ | **✔**​ | **​A** |
|  | Fornecer opinião honesta e construtiva para ajudar os ECRs a solucionar problemas & aperfeiçoar ideias | **-** | **✔**​ | **✔**​ | **✔**​ | **✔**​ | **✔**​ | **✔**​ |
|  | Use atividades de melhoria de investigação para melhorar projetos existentes | **$/-** | **✔**​ | **✔**​ | **✔**​ | **✔**​ | **✔**​ | **✔**​ |
|  | Trabalhe com os ECRs para garantir que as melhorias são sustentáveis ao integrar as alterações nos procedimentos operacionais padrão ou nos manuais de laboratório | **-** | **✔**​ | **✔**​ | **✔**​ | **✔**​ | **✔**​ | **✔**​ |
|  | Aumentar a visibilidade dos esforços liderados pelos ECRs para melhorar a ciência; dar oportunidades aos ECRs de compartilhar as suas atividades de melhoria de investigação com os outros | **$/-** | **✔**​ | **✔**​ | **✔**​ | **✔**​ | **✔**​ | **✔** |
| Proteger esforços para apoiar ECRs marginalizados* | Promova uma cultura de diversidade & inclusão | **-** | **✔**​ | **✔**​ | **✔**​ | **✔**​ | **✔**​ | **✔**​ |
|  | Identifique & elimine as barreiras à participação plena | **$/-** | **✔**​ | **✔**​ | **✔**​ | **✔**​ | **✔**​ | **✔**​ |
|  | Promulgar políticas para garantir a representação de grupos marginalizados em cargos de liderança | **$/-** | **✔**​ | **✔**​ | **✔**​ | **✔**​ | **✔**​ | **​A** |
| Apoiar iniciativas globais para melhoria da cultura e prática de investigação | Organize conferências virtuais ou híbridas e eventos de networking, ou use formatos que permitam a participação assíncrona (por exemplo, brainstorming virtual) | **$/-** | ​ | **✔**​ | **✔**​ | **✔**​ | **✔**​ | **​A** |
|  | Oferecer subsídios de melhoria científica para ECRs em países ou comunidades com financiamento de investigação limitado | **$** | ​ | **✔**​ | ​ | **✔** | ​ | **​A** |
|  | Cientistas de países onde a investigação é comparativamente bem financiada devem identificar oportunidades para ampliar os esforços daqueles com menos recursos | **$/-** | **✔**​ | **✔**​ | **✔**​ | **✔**​ | **✔**​ | **✔**​ |
|  | Incluir ECRs de países com financiamento de investigação limitado quando adicionar representantes aos comités. Garantir que essa diversidade também é refletida entre os membros do comité não ECR. | **$/-** | ​ | ​ | **✔**​ | **✔**​ | **✔**​ | **​A** |

***Tabela S8.*** ***Ações que organizações e indivíduos podem adotar para apoiar os ECRs na melhoria da publicação científica e da cultura de investigação***

*As marcas de seleção indicam ações específicas que indivíduos ou organizações podem realizar para apoiar e amplificar as atividades de ECRs para melhorar a ciência. A letra A denota ações que aliados, supervisores ou mentores podem defender como parte dos cargos que ocupam dentro de uma organização.*

** Indivíduos e organizações devem adotar as três recomendações abaixo em todos os empreendimentos científicos, incluindo no seu trabalho científico e ao implementar quaisquer ações descritas nesta tabela. Consulte os recursos atuais de melhores práticas, pois as práticas de diversidade, equidade e inclusão dependem do contexto e evoluem ao longo do tempo.*
