# Supplementary material for: Recommendations for empowering early career researchers to improve research culture and practice
Source: PLoS Biol. 2022 Jul 7;20(7):e3001680. doi: 10.1371/journal.pbio.3001680 (PMC9295962; doi:10.1371/journal.pbio.3001680)
Supplement: S9 Table — Aktivnosti koje organizacije i pojedinci mogu da sprovode kako bi podržali mlade istraživače u unapređivanju naučnog izdavaštva i nauke. Kvačica označava aktivnosti koje pojedinci ili organizacije mogu da sprovedu da bi podržali i uvećali značaj aktivnosti mladih istraživača za unapređivanje nauke. Slovo A označava aktivnosti koje saveznici, supervizori ili mentori mogu da zagovaraju u okviru svojih pozicija unutar organizacija.* Pojedinci i organizacije treba da usvoje tri preporuke u svim svojim naučnim aktivnostima, uključujući naučni rad i kada se primenjuju aktivnosti opisane u ovoj tabeli. Potrebno je konsultovati trenutne resurse o najboljim praksama pošto se prakse diverziteta, jednakosti i inkluzije menjaju tokom vremena i zavise od konteksta. (DOCX) [file pbio.3001680.s018.docx]

**Preporuke za osnaživanje mladih istraživača radi unapređenja istraživačke kulture i prakse**

| Preporuka | **Vid podrške** | **Trošak** | **Institucije i odeljenja** | **Agencije za finansiranje** | **Naučni časopisi i izdavači** | **Naučna udruženja** | **Udruženja mladih istraživača** | **Saveznici, supervizori i mentori** |
| --- | --- | --- | --- | --- | --- | --- | --- | --- |
| Obezbediti putanju za napredovanje u karijeri putem nagrađivanja i davanja podsticaja aktivnostima za unapređenje nauke | Kreiranje radnih mesta za meta-istraživače i druge koji rade na unapređenju nauke | **$** | **✔**​ | **✔**​ | **✔**​ | **✔**​ | ​ | **​A** |
|  | ​Nagrađivanje za aktivnosti za unapređenje nauke kroz zapošljavanje i unapređivanje | **-** | **✔**​ | **✔**​ | **✔**​ | **✔**​ | ​ | **​A** |
|  | ​Uključivanje aktivnosti za unapređenje nauke u evaluacije grantova za treninge | **-** | **✔**​ | **✔**​ | ​ | ​ | ​ | **​A** |
|  | Objavljivanje meta-istraživanja i radova na temu unapređenja nauke (idealno s otvorenim pristupom)​ | **$/-** | ​ | ​ | **✔**​ | ​ | ​ | **​A** |
|  | Dodeljivanje nagrada za aktivnosti za unapređenje nauke | **$/-** | **✔**​ | **✔**​ | **✔**​ | **✔**​ | **✔** | **​A** |
| Uključiti mlade istraživače u procese donošanja odluka | Kreiranje svetodavnih grupa čiji sastavni deo čine mladi istraživači i održavanje dijaloga sa donosiocima odluka | **$/-** | **✔**​ | **✔**​ | **✔**​ | **✔**​ | ​ | **​A** |
|  | Uključivanje mladih istraživača u naučne komitete, kreiranje gostoljubive atmofere i podrške | **$/-** | **✔**​ | **✔**​ | **✔**​ | **✔**​ | ​ | **​A** |
|  | Razmatranje kombinovanja savetodavnih grupa mladih istraživača sa predstavnicima mladih istraživača u komitetima | **$/-** | **✔**​ | **✔**​ | **✔**​ | **✔**​ | ​ | **​A** |
| Mladim istraživačima s kvalifikacijama za unapređivanje istraživanja, obezbediti sredstva, finansiranje i vreme za bavljenje unapređivanjem istraživačke kulture i prakse | Kreiranje grantova za unapređivanje nauke, tako da mladi istraživači mogu da ispune uslove prijavljivanja | **$** | **✔**​ | **✔**​ | **✔**​ | **✔**​ | ​ | **​A** |
|  | Kreiranje malih grantova za mlade istraživače koji imaju ideje za unapređivanje naučnog izdavaštva | **$** | ​ | **✔**​ | **✔**​ | **✔**​ | ​ | **​A** |
|  | Obezveđivanje logističke ili administrativne podrške za inicijative mladih istraživača (npr. u vidu menadžera udruženja)​ | **$** | **✔**​ | **✔**​ | **✔**​ | **✔**​ | ​ | **​A** |
|  | Objavljivanje programa i rezultata koji su od značaja za udruženja mladih istraživača | **$/-** | **✔**​ | **✔**​ | **✔**​ | **✔**​ | **✔** | **✔**​ |
|  | Kreiranje grantova koji mladim istraživačima obezbeđuju vreme namenjeno za aktivnosti za unapređenje istraživanja | **$** | **✔**​ | **✔**​ | ​ | **✔**​ | ​ | **​A** |
|  | Ohrabrivanje mladih istraživača da aktivnosti za unapređenje nauke uključe u svoje planove za razvoj karijere | **-** | **✔**​ | **✔**​ | ​ | **✔**​ | ​ | **✔**​ |
| Prepoznati ekspertizu mladih istraživača i naglasiti njihove napore za unapređivanje nauke | Kreiranje (onlajn) zajednica za mlade istraživače koji rade na unapređivanju naučne kulture i prakse | **$/-** | **✔**​ | **✔**​ | **✔**​ | **✔**​ | **✔**​ | **​A** |
|  | Trening naučnika po pitanju veština potrebnih za unapređivanje nauke na pojedinačnom i sistemskom nivou | **$/-** | **✔**​ | **✔**​ | **✔**​ | **✔**​ | **✔**​ | **​A** |
|  | Obezbeđivanje pravedne i konstruktivne povratne poruke kako bi se mladim istraživačima pomoglo da reše probleme i poboljšaju svoje ideje | **-** | **✔**​ | **✔**​ | **✔**​ | **✔**​ | **✔**​ | **✔**​ |
|  | Primena aktivnosti za unapređivanje istraživanja na postojeće projekte | **$/-** | **✔**​ | **✔**​ | **✔**​ | **✔**​ | **✔**​ | **✔**​ |
|  | Rad sa mladim istraživačima kako bi se osiguralo da su poboljšanja održiva i nakon što mladi istraživači nastave sa svojim radom; Integrisanje promena u standardne radne procedure ili laboratorijska uputstva | **-** | **✔**​ | **✔**​ | **✔**​ | **✔**​ | **✔**​ | **✔**​ |
|  | Poboljšavanje vidljivosti napora mladih istraživača za unapređivanje nauke, obezbeđivanje prilika da mladi istraživači podele svoje aktivnosti za unapređivanje istraživanja sa drugima | **$/-** | **✔**​ | **✔**​ | **✔**​ | **✔**​ | **✔**​ | **✔** |
| Pružiti podršku mladim istraživačima iz marginalizovanih grupa​* | Negovanje kulture diverziteta i inkluzije | **-** | **✔**​ | **✔**​ | **✔**​ | **✔**​ | **✔**​ | **✔**​ |
|  | Identifikovanje i uklanjanje prepreka za puno učešće | **$/-** | **✔**​ | **✔**​ | **✔**​ | **✔**​ | **✔**​ | **✔**​ |
|  | Primena smernica kako bi marginalizovane grupe bile predstavljene na vodećim pozicijama | **$/-** | **✔**​ | **✔**​ | **✔**​ | **✔**​ | **✔**​ | **​A** |
| Podržati globalne inicijative za unapređivanje istraživačke kulture i prakse | Organizacija virtuelnih ili hibridnih konferencija i *networking* događaja, ili upotreba formata koji omogućavaju asinhrono učešće (npr. virtuelni *brainstorming*)​ | **$/-** | ​ | **✔**​ | **✔**​ | **✔**​ | **✔**​ | **​A** |
|  | Obezbeđivanje grantova za unapređenje istraživanja mladim istraživačima u zemljama ili zajednicama koje raspolažu ograničenim sredstvima za finansiranje istraživanja | **$** | ​ | **✔**​ | ​ | **✔** | ​ | **​A** |
|  | Naučnici iz zemalja u kojima je istraživanje relativno dobro finansirano treba da identifikuje prilike za povećanje napora onih istraživača koji imaju manje sredstava | **$/-** | **✔**​ | **✔**​ | **✔**​ | **✔**​ | **✔**​ | **✔**​ |
|  | Prilikom uključivanja predstavnika mladih istraživača u komitete, treba uključiti mlade istraživače iz zemalja sa ograničenim sredstvia za finansiranje istraživanja. Istovremeno treba osigurati diverzitet među članovima komiteteta koji nisu mladi istraživači. | **$/-** | ​ | ​ | **✔**​ | **✔**​ | **✔**​ | **​A** |

***Tabela S9.*** ***Aktivnosti koje organizacije i pojedinci mogu da sprovode kako bi podržali mlade istraživače u unapređivanju naučnog izdavaštva i nauke.***

*Kvačica označava aktivnosti koje pojedinci ili organizacije mogu da sprovedu da bi podržali i uvećali značaj aktivnosti mladih istraživača za unapređivanje nauke. Slovo A označava aktivnosti koje saveznici, supervizori ili mentori mogu da zagovaraju u okviru svojih pozicija unutar organizacija.*

** Pojedinci i organizacije treba da usvoje tri preporuke u svim svojim naučnim aktivnostima, uključujući naučni rad i kada se primenjuju aktivnosti opisane u ovoj tabeli. Potrebno je konsultovati trenutne resurse o najboljim praksama pošto se prakse diverziteta, jednakosti i inkluzije menjaju tokom vremena i zavise od konteksta.*
