# Supplementary material for: Recommendations for empowering early career researchers to improve research culture and practice
Source: PLoS Biol. 2022 Jul 7;20(7):e3001680. doi: 10.1371/journal.pbio.3001680 (PMC9295962; doi:10.1371/journal.pbio.3001680)
Supplement: S10 Table — Acciones que las organizaciones y los individuos pueden tomar para apoyar a los ECR en la mejora de la publicación científica y la cultura de investigación. Las marcas de verificación indican acciones específicas que los individuos u organizaciones pueden tomar para apoyar y ampliar las actividades de ECR para mejorar la ciencia. La letra A denota acciones por las que los aliados, supervisores o mentores pueden abogar como parte de los puestos que ocupan dentro de una organización.* Las personas y organizaciones deben adoptar las tres recomendaciones en todos los esfuerzos científicos, incluido su trabajo científico y al implementar cualquier acción descrita en esta tabla. Consulte los recursos de mejores prácticas actuales, ya que las prácticas de diversidad, equidad e inclusión dependen del contexto y evolucionan con el tiempo. (DOCX) [file pbio.3001680.s019.docx]

**Recomendaciones para Empoderar a los Investigadores de Carrera Temprana orientada a Mejorar la Cultura y la Práctica en la Investigación**

| **Recomendación** | **Acciones de Apoyo** | **Costo** | **Instituciones y Departamentos** | **Agencias de Financiamiento** | **Revistas y Editores** | **Sociedades Científicas** | **Comunidades de pares ECR** | **Aliados, Supervisores y Mentores** |
| --- | --- | --- | --- | --- | --- | --- | --- | --- |
| Proporcionar un camino para el progreso profesional al recompensar e incentivar las actividades de mejora de la ciencia. | Crear posiciones para meta-investigadores y otros que trabajan para mejorar la ciencia | **$** | **✔** | **✔** | **✔** | **✔** | ​ | **​A** |
|  | Recompensar las actividades de mejora científica en la contratación y promoción | **-** | **✔** | **✔** | **✔** | **✔** | ​ | **​A** |
|  | Incorporar actividades de mejora de la ciencia en las evaluaciones de becas de capacitación. | **-** | **✔** | **✔** | ​ | ​ | ​ | **​A** |
|  | Publicar artículos de meta-investigación y mejora de la ciencia (idealmente de acceso abierto ) | **$/-** | ​ | ​ | **✔** | ​ | ​ | **​A** |
|  | Ofrecer premios para actividades de mejora de la ciencia. | **$/-** | **✔** | **✔** | **✔** | **✔** | **✔** | **​A** |
| Integrar los ECR en los procesos de toma de decisiones | Crear grupos asesores compuestos por ECR y mantener un diálogo sólido con los órganos de toma de decisiones | **$/-** | **✔** | **✔** | **✔** | **✔** | ​ | **​A** |
|  | Incluir representantes de ECR en los comités científicos; crear un ambiente acogedor y de apoyo | **$/-** | **✔** | **✔** | **✔** | **✔** | ​ | **​A** |
|  | Considerar combinar grupos asesores de ECR con representantes de ECR en comités | **$/-** | **✔** | **✔** | **✔** | **✔** | ​ | **​A** |
| Proporcionar a los ECR expertos en la mejora de la investigación recursos, financiación y tiempo protegido para mejorar la cultura y la práctica en la investigación. | Crear subvenciones para la mejora de las ciencias; Asegurarse de que los ECR sean elegibles para aplicar | **$** | **✔** | **✔** | **✔** | **✔** | ​ | **​A** |
|  | Crear pequeñas subvenciones para ECR que tengan ideas sobre cómo mejorar la publicación científica | **$** | ​ | **✔** | **✔** | **✔** | ​ | **​A** |
|  | Ofrecer apoyo logístico o administrativo para las iniciativas de ECR (por ejemplo, un administrador de la comunidad ) | **$** | **✔** | **✔** | **✔** | **✔** | ​ | **​A** |
|  | Publicitar programas o resultados valiosos para la comunidad ECR | **$/-** | **✔** | **✔** | **✔** | **✔** | **✔** | **✔**​ |
|  | Ofrecer subvenciones que brinden a las ECR tiempo protegido para actividades de mejora de la investigación | **$** | **✔** | **✔** | ​ | **✔** | ​ | **​A** |
|  | Alentar a los ECR a incorporar actividades de mejora científica en los planes de desarrollo profesional. | **-** | **✔** | **✔** | ​ | **✔** | ​ | **✔**​ |
| Reconocer la experiencia de los ECR y ampliar sus esfuerzos para mejorar la ciencia  ​  ​ | Crear comunidades (en línea) para ECR que trabajen para mejorar la cultura y las prácticas científicas | **$/-** | **✔** | **✔** | **✔** | **✔** | **✔** | **​A** |
|  | Capacitar a los científicos en las habilidades necesarias para mejorar la ciencia a nivel individual y sistémico | **$/-** | **✔** | **✔** | **✔** | **✔** | **✔** | **​A** |
|  | Proporcionar comentarios honestos y constructivos para ayudar a los ECR a solucionar problemas y refinar ideas | **-** | **✔** | **✔** | **✔** | **✔** | **✔** | **✔**​ |
|  | Utilizar actividades de mejora de la investigación para mejorar los proyectos existentes | **$/-** | **✔** | **✔** | **✔** | **✔** | **✔** | **✔**​ |
|  | Trabajar con los ECR para garantizar que las mejoras sean sostenibles después de que los ECR avancen integrando los cambios en los procedimientos operativos estándar o en los manuales de laboratorio. | **-** | **✔** | **✔** | **✔** | **✔** | **✔** | **✔**​ |
|  | Aumentar la visibilidad de los esfuerzos liderados por ECR para mejorar la ciencia; brindar a los ECR oportunidades para compartir sus actividades de mejora de la investigación con otros | **$/-** | **✔** | **✔** | **✔** | **✔** | **✔** | **✔** |
| Defender los esfuerzos para apoyar a los ECR marginados* | Fomentar una cultura de diversidad e inclusión | **-** | **✔** | **✔** | **✔** | **✔** | **✔** | **✔**​ |
|  | Identificar y eliminar las barreras para la plena participación | **$/-** | **✔** | **✔** | **✔** | **✔** | **✔** | **✔**​ |
|  | Promulgar políticas para garantizar la representación de los grupos marginados en puestos de liderazgo | **$/-** | **✔** | **✔** | **✔** | **✔** | **✔** | **​A** |
| Apoyar iniciativas globales para mejorar la cultura y la práctica de la investigación | Organizar conferencias virtuales o híbridas y eventos de networking, o usar formatos que permitan la participación asíncrona (por ejemplo, lluvia de ideas virtual) | **$/-** | ​ | **✔** | **✔** | **✔** | **✔** | **​A** |
|  | Ofrecer subvenciones para la mejora de la investigación para ECR en países o comunidades con financiación limitada para la investigación | **$** | ​ | **✔** | ​ | **✔** | ​ | **​A** |
|  | Los científicos de países donde la investigación está comparativamente bien financiada deben identificar oportunidades para ampliar los esfuerzos de aquellos con menos recursos. | **$/-** | **✔** | **✔** | **✔** | **✔** | **✔** | **✔**​ |
|  | Al agregar representantes de ECR a los comités, incluir ECR de países con financiamiento de investigación limitado. Asegurarse de que esta diversidad también se refleje entre los miembros del comité que no son ECR. | **$/-** | ​ | ​ | **✔** | **✔** | **✔** | **​A** |

***Tabla S10.*** ***Acciones que las organizaciones y los individuos pueden tomar para apoyar a los ECR en la mejora de la publicación científica y la cultura de investigación.***

*Las marcas de verificación indican acciones específicas que los individuos u organizaciones pueden tomar para apoyar y ampliar las actividades de ECR para mejorar la ciencia. La letra A denota acciones por las que los aliados, supervisores o mentores pueden abogar como parte de los puestos que ocupan dentro de una organización.*

** Las personas y organizaciones deben adoptar las tres recomendaciones en todos los esfuerzos científicos, incluido su trabajo científico y al implementar cualquier acción descrita en esta tabla. Consulte los recursos de mejores prácticas actuales, ya que las prácticas de diversidad, equidad e inclusión dependen del contexto y evolucionan con el tiempo.*
